# Cross-Kingdom Analysis of Diversity, Evolutionary History, and Site Selection within the Eukaryotic Macrophage Migration Inhibitory Factor Superfamily

**DOI:** 10.3390/genes10100740

**Published:** 2019-09-24

**Authors:** Claire Michelet, Etienne G. J. Danchin, Maelle Jaouannet, Jürgen Bernhagen, Ralph Panstruga, Karl-Heinz Kogel, Harald Keller, Christine Coustau

**Affiliations:** 1 Institut Sophia Agrobiotech, Université Côte d’Azur, INRA, CNRS, 400 Route des Chappes, F-06903 Sophia Antipolis, France; ewilan06@hotmail.fr (C.M.); etienne.danchin@inra.fr (E.G.J.D.); maelle.jaouannet@inra.fr (M.J.); harald.keller@inra.fr (H.K.); 2 Department of Vascular Biology, Institute for Stroke and Dementia Research (ISD), Klinikum der Universität München (KUM), Ludwig-Maximilians-University (LMU), D-81377 Munich, Germany; Juergen.Bernhagen@med.uni-muenchen.de; 3 Unit of Plant Molecular Cell Biology, Institute for Biology I, RWTH Aachen University, Worringerweg 1, D-52056 Aachen, Germany; panstruga@bio1.rwth-aachen.de; 4 Department of Phytopathology, Center of BioSystems, Land Use and Nutrition (iFZ), Justus Liebig University (JLU), D-35392 Giessen, Germany; karl-heinz.kogel@agrar.uni-giessen.de

**Keywords:** macrophage migration inhibitory factor, host-parasite interactions, innate immunity, phylogenetic reconstructions, eukaryotes

## Abstract

Macrophage migration inhibitory factors (MIF) are multifunctional proteins regulating major processes in mammals, including activation of innate immune responses. MIF proteins also play a role in innate immunity of invertebrate organisms or serve as virulence factors in parasitic organisms, raising the question of their evolutionary history. We performed a broad survey of MIF presence or absence and evolutionary relationships across 803 species of plants, fungi, protists, and animals, and explored a potential relation with the taxonomic status, the ecology, and the lifestyle of individual species. We show that MIF evolutionary history in eukaryotes is complex, involving probable ancestral duplications, multiple gene losses and recent clade-specific re-duplications. Intriguingly, MIFs seem to be essential and highly conserved with many sites under purifying selection in some kingdoms (e.g., plants), while in other kingdoms they appear more dispensable (e.g., in fungi) or present in several diverged variants (e.g., insects, nematodes), suggesting potential neofunctionalizations within the protein superfamily.

## 1. Introduction

Macrophage migration inhibitory factors (MIFs) are pivotal pro-inflammatory cytokines/chemokines involved in infectious and immune diseases in humans, such as septic shock, colitis, malaria, rheumatoid arthritis, and atherosclerosis, as well as tumorigenesis [1,2,3,4,5]. They contribute to important physiological functions including control of the cell cycle, sensing of pathogen stimuli, activation of innate defense responses, recruitment and homeostasis of various immune cells, and prevention of p53-mediated apoptosis [6]. Despite a limited molecular mass and a lack of clear modular domains, human MIF contains several sequence motifs including three potential catalytic sites that provide this protein with a surprising functional complexity [2,7]. Multi-functionality may also be explained by the fact that the protein is remarkably amenable to interactions with a number of target proteins, despite its overall architectural rigidity [8]. For example, human MIF appears to be involved, directly or indirectly, in 49 signaling reactions, thus evidencing the extraordinary functional complexity of the protein [9].

The vertebrate MIF protein family is composed of the actual MIF and D-DT (D-dopachrome tautomerase), which are evolutionarily ancient proteins since MIF/D-DT-like genes exist in bacteria [10]. In some vertebrate parasites such as protozoa, nematodes, or ticks, MIF/D-DT proteins are secreted and participate in the modulation of the immune system of the vertebrate host [10,11]. Intriguingly, free-living invertebrate species also seem to require MIF/D-DT to mount their immune responses, although most of the MIF membrane-localized partner molecules (‘the MIF receptors’) identified in mammals are missing. For example, in the gastropod snail, *Biomphalaria glabrata*, MIF contributes to the activation of immune responses, promotes immune cell proliferation, and inhibits p53-mediated apoptosis [12]. These functions seem to be general features of MIF in mollusks [13], but also occur in invertebrates such as crustaceans and echinoderms [14]. Even more intriguing were the findings that plants possess MIF genes (named MDL for MIF/DDT-like) [15], and that aphids secrete a MIF protein upon feeding to manipulate plant immunity [16]. Such results were unexpected since plants are not only missing the known MIF partner molecules but also most of the known MIF-related signaling pathways. Although the plant immune system shares some similarities with innate immunity in animals, such as the occurrence of pattern-recognition receptors (PRR), nitric oxide signaling, mitogen-activated protein kinase cascades and an oxidative burst, it mainly involves three major plant-specific hormonal pathways, namely the salicylic acid, jasmonic acid, and ethylene signaling pathways [17]. In addition, some essential compounds of the animal immune system are missing in plants, such as a circulatory system, specialized immune cells, and prototypical immune cell receptors of the G protein-coupled receptor (GPCR) class. Therefore, the roles that both plant and insect MIFs play during host-parasite interactions and how they may interact with plant signaling pathways remain unclear and deserve dedicated studies [15,16].

Our current knowledge on non-mammalian MIFs raises questions on the evolutionary history of these proteins, and on a putative diversification of structure affecting their function. First, the pattern of diversification, expansion or loss of *MIF* genes across eukaryotic kingdoms is presently unknown. While MIF proteins are diversified and functionally essential in some species such as aphids, they are missing in other insects such as *Drosophila*, therefore suggesting a complex evolutionary history with differential losses and duplications [18]. The first goal of this study was to test the hypothesis of a complex evolutionary history of MIFs with differential losses and duplications possibly related to biological traits such as the taxonomic status, the ecology, and the lifestyle of individual species. To address this question, we performed a broad survey of the presence or absence of MIF across 803 species of plants, fungi, protists, and animals. We assessed the number of MIF of individual species and explored a potential relation between MIF number and the taxonomic status, the ecology, and the lifestyles. Second, we aimed at further exploring the evolutionary history of this protein family through a general phylogenetic analysis across kingdoms. Third, because MIF proteins, when present, can be essential immune regulators involved in host survival or parasite virulence, or possibly have yet unknown functions, we hypothesized that differential selection pressures may have driven the evolution of MIF sequences according to the free-living versus parasitic lifestyles of the respective species. We therefore investigated the structure/function relationship by analyzing site selection on sequences from selected phyla including free-living species and parasites of plants and animals. Accordingly, the conservation or diversification of specific amino acid sites or motifs of MIF proteins across kingdoms were examined as they might be informative on the earliest MIF function(s) and give insights into the evolution of MIF functions.

## 2. Materials and Methods

### 2.1. In Silico Identification of MIF Proteins

Typical MIF protein sequences (i.e., small proteins composed of a single MIF domain) were searched across eukaryotes, with a special emphasis on phyla including both, free-living species, and parasites of plants and animals. The species belonging to protists, fungi, metazoan and plants that were extensively searched for MIF sequences are listed in the Supplementary Table S1. To identify typical MIF proteins, we performed manual BLASTP [19] searches, systematically using as queries twelve published MIF sequences from six phylogenetically distant species representing six main clades of the eukaryotic tree of life: (i) the vertebrate human MIF family members (MIF and D-DT/MIF-2), (ii) the metazoan mollusc *Biomphalaria glabrata*, (iii) the metazoan nematode *Ancylostoma caninum* [20], (iv) the *Alveolata Plasmodium berghei*, (v) the *Excavata Leishmania major*, (vi) the plant *Arabidopsis thaliana*. Accession numbers of these queries are provided in the Supplementary Table S2.

These twelve proteins were first used as queries for BLASTP searches against the general sequence libraries Ensembl [21] and the National Center for Biotechnology Information (NCBI) [22] nr library. We also searched taxon-specific libraries: Archives for fungal sequences (Joint Genome Institute (JGI [23]) and FungiDB [24]; *Diptera* (Flybase [25], *Hymenoptera* (HymenopteraMine [26]), and aphids (Aphidbase [27]); nematodes (wormbase [28] and wormbase parasite [29]), human parasites (GeneDB [30]). MIF protein sequences were also searched in publicly available databases from genome sequencing consortia: *Cyanophora paradoxa* (*Cyanophora* genome project [31]), *Cryptosporidium hominis* (CryptoDB [32]), *Globodera pallida* (in the Sanger server at http://www.sanger.ac.uk/cgi-bin/blast/submitblast/g_pallida), *Pristionchus pacificus* (at http://www.pristionchus.org/blast/) and *Meloidogyne* species (at http://meloidogyne.inra.fr). All the BLASTP searches were performed using the scoring matrix BLOSUM62 [33], no low complexity filtering, a cut-off E-value of 10^−10^ and coverage >90% of the query length. The BLAST hits of the 12 queries against the libraries and databases were collected and redundancy at 100% identity between the hits was eliminated.

To confirm the diversity and abundance of canonical (typical) MIFs in the species of interest listed in the Supplementary Dataset 1, we also performed TBLASTN searches using the same 12 queries (scoring matrix BLOSUM62, SEG low complexity filtering, cut-off E-value of 10^−10^, and coverage >90%) against expressed sequence tag (EST) libraries, whole genome shotgun contigs (WGS), or transcriptome shotgun assemblies (TSA) at the NCBI. All the protein sequences retained after the BLAST searches were checked for the presence of the characteristic MIF domain (IPR014347 and/or IPR001398) by manual scan against Interproscan [34], (online with default parameters) and were confirmed to present the typical MIF domain.

### 2.2. Classification of Species Taxonomy, Ecology, and Lifestyle

Information concerning the cited species were retrieved from the NCBI taxonomy browser (https://www.ncbi.nlm.nih.gov/taxonomy), the Encyclopedia of Life (https://eol.org/pages/), and from links and references cited therein. With respect to the ecology of species, we took into account whether the general environment is terrestrial, freshwater, or marine. We examined the lifestyle of species regarding the mode of feeding, and classified organisms as free-living autotrophs, heterotrophs, and animal- or plant-parasites. To simplify, we considered as ‘parasite’ all species engaged in a sustained interaction with a host, including, for example, mutualistic symbionts or species classically referred to as ‘pathogens’. The rationale was that these species share, to some extent, evolutionarily important constraints such as establishing a sustained molecular dialog with the host and evading or manipulating host immune responses, at least at an early stage of infection.

### 2.3. Statistical Analyses

Statistical analyses were performed with the R package [35], version 3.1.3. The Multiple Correspondence Analysis (MCA [36]) was performed using the function ‘dudi.acm’ present in the package ‘ade4′ [37]. The potential links between the number of MIF sequences present in each species were analyzed as response variable of a generalized linear regression model (GLM [38]) using as predictors the taxonomy, the environment and the lifestyle (free-living or parasite). A quasi-Poisson distribution was used to account for under-dispersion in the data, and interactions were not included. Analysis of deviance was performed to check for the effect of the three predictors. In a second time, another identical GLM model was fitted for only four major phyla, with lifestyle as a predictor. In both cases zero inflation was tested for by using the zeroinfl function (package pcls [39]) and was not detected.

### 2.4. Multiple Sequence Alignments and Phylogenetic Reconstructions

MIF protein alignments were performed using MUSCLE [40] with default parameters in Jalview version 2.9 [41] and examined in Jalview to detect and manually curate incomplete protein sequence predictions, sequencing errors and poorly aligned sequences. The alignments were automatically cleaned using trimAl [42] on gappyout mode with standard settings. Sequences containing less than 85% of amino acid overlaps with the rest of the alignment were removed. The 18S ribosomal RNA (rRNA) sequences were aligned using the specific SINA aligner [43] (SILVA Incremental Aligner) with default parameters. This tool is optimized for 18S sequences and is available in the SILVA databases [44] (https://www.arb-silva.de/).

For phylogenetic reconstructions, we used the two complementary methods of maximum likelihood (ML) analysis [33] and Bayesian inference (BI) of phylogeny [45]. For both methods, the best amino acid substitution models in the MIF phylogeny were estimated from the multiple alignments using ProtTest [46] version 3. The Wag model (without inclusion of a proportion of invariable sites or γ distribution of the rates of evolution) was systematically estimated to be the fittest for the MIF analyses. The ML analysis was performed using the PhyML [47] method implemented in the SeaView [48] (version 4) software with the following settings: amino acid frequencies (optimized), proposition of invariable sites and number of substitution rate categories estimated from the Datasets. BIONJ was chosen to create the starting tree and the best method between the NNI (Nearest Neighbor Interchanges) and SPR (Subtree Pruning and Regrafting) tree improvement methods were used to estimate the best topology, and the tree topology optimization option was chosen. Branch support values were calculated using an approximate likelihood-ratio test (aLRT). Bayesian phylogenetic analysis was performed using MrBayes [49] version 3.2.6 and four independent analyses starting from different random trees. The Markov chain Monte Carlo (MCMC) simulations were automatically stopped after average deviation of split frequencies reached a value <0.01 (this was reached after 10,500,000 simulations). For final phylogenetic tree reconstruction (sumt) and probabilities analysis (sump), 25% of the trees were ‘burnt’. To root the MIF phylogenetic reconstruction, we used the most closely related non-eukaryotic MIF sequences from different bacteria as an outgroup: *Synechococcus sp*., *Thioalkalivibrio nitratireducens*, *Thioalkalivibrio paradoxus*, *Oscillatoria_sp*. and *Microcoleus sp*. (Genbank accession number WP_011360252.1, WP_015260126.1, WP_006746032.1, WP_017717428.1 and WP_015181735.1, respectively).

As a reference taxonomic information, the species phylogenetic trees were reconstructed *de novo* on the 18S rRNA of species from selected phyla (e.g., *Alveolata* or insects), using the *Arabidopsis thaliana* sequence as an outgroup. For the 18S rRNA, the best nucleotide substitution models were estimated for alignments using JModelTest [50] with default settings and the starting tree. The best method between the NNI (Nearest Neighbor Interchanges) and SPR (Subtree Pruning and Regrafting) tree improvement methods was used to create the starting tree. For illustrative purposes, MIF and species trees were colored in a similar way, based on broad phylogenetic taxa (plants, fungi, *Alveolata*, metazoan, etc.). When appropriate for result interpretation, the public knowledge database TimeTree [51] (available at http://www.timetree.org/) was used to provide an estimated divergence time between two taxa, based on published time estimates [51].

### 2.5. Analysis of Purifying/Diversifying Selection

Conservation or diversification of particular amino acids and sequence motifs within MIF sequences was investigated on sequences from *Alveolata* (parasites of animals), nematodes (plant and animal parasites considered separately), oomycetes (plant parasites), aphids (plant parasites) and plants. *Alveolata* sequences encompassed four *Eimeria* species, ten *Plasmodium* species as well as *Toxoplasma gondii*, *Hammondi hammondi* and *Neospora caninum* (17 sequences). Oomycetes were represented by all MIF sequences from *Phytophthora sojae, P. parasitica*, and *P. infestans* (nine sequences). For nematode parasites of animals, we selected the MIF sequences from *Ostertagia ostertagi, Ascaris suum, Onchocerca volvulus, Brugia malayi, Loa loa, Trichinella spiralis, and Ancylostoma ceylanicum* (seven sequences). For plant-parasitic nematodes, we used the sequences of all MIF family members from *Bursaphelenchus xylophilus*, *Meloidogyne hapla*, and *Meloidogyne incognita* (six sequences). Aphids were represented by all MIF sequences from *Acyrthosiphon pisum*, *Myzus persicae*, *Rhopalosiphum maidis* and *Rhopalosiphum padi* (13 sequences). Finally, the analysis of plant MIFs was performed on sequences from seven monoliophyte (ferns) species, four chlorophyte (green algae) species, two bryophyte (moss) species, one marchantiophyte (liverwort), one spermatophyte, five monocotyledonous species and five eudicotyledonous species (total of 47 sequences). Multiple sequence alignments of MIF proteins were converted into codon alignments via PAL2NAL [52] by providing the corresponding coding sequences (CDS). The existence of purifying (negative) or diversifying (positive) site selection was investigated by a combination of complementary models implemented in the Selecton [53] and Datamonkey [54] online servers. Selecton uses a BI approach to calculate the ratio between non-synonymous and synonymous substitutions. Analyses with the Selecton tool were performed with the positive selection enabled model (M8, β + w ≥ 1), new tree and the default settings. The null hypothesis was tested using the M8a, β + w = 1 and the M7, β models.

For the calculations we adopted the following five models in the Datamonkey server: (1) The single likelihood ancestor counting (SLAC), (2) The fixed effect likelihood (FEL), (3) The Random Effect Likelyhood (REL), (4) the mixed-effects model of evolution [55] (MEME), which combines the fixed and random effects to identify instances of both episodic diversifying selection and pervasive positive selection at the individual branch site level, and (5) the fast unconstrained Bayesian approximation (FUBAR) using Markov Chain Monte Carlo routine. Analyses on the basis of FEL, REL, SLAC, FUBAR, and MEME were conducted with the following settings: global dN/dS estimated with CI (likelihood profile based on 95% confidence interval), the significance level was set at 0.1 and ambiguities were resolved. The substitution model selection was determined by the automatic substitution model selection tool included in the Datamonkey server. The trees required by FEL, REL, MEME, FUBAR, and Selecton software were reconstructed using the ML and BI methods. After verifying that the resulting trees were similar, we chose the ML trees for site selection analyses.

## 3. Results

### 3.1. MIF Presence and Number Across Eukaryotic Kingdoms

To link the abundance of MIF protein sequences with the taxonomy and ecology of species across eukaryotic kingdoms, we first investigated the presence of MIFs in species from protists, fungi, animals, and plants. In case of the former three taxonomic groups, we focused on phyla comprising both free-living species and parasites of plants and animals. In total, we analyzed 803 species for the presence and number of MIF sequences in relation to information on their taxonomy, their environment (fresh water, marine, or terrestrial) and their lifestyle (free-living heterotrophic or autotrophic species, plant parasite or animal parasite; see Materials and Methods). In addition, we considered the presence or absence of a sequenced genome and the number of available ESTs when no sequenced genome was available, thus gathering together data that provided a solid framework for our study (Supplementary Dataset 1). Depending on the availability of a sequenced genome and on the quality of assembly and annotation, comprehensive identification of gene copy number was not always possible. We thus do not report here the number of *MIF* gene copies, but the number of distinct MIF protein sequences retrieved after extensive BLAST searches. The term ‘MIF number’ used throughout the manuscript therefore refers to the number of MIF proteins identified at the time of the study.

**Figure 1:**
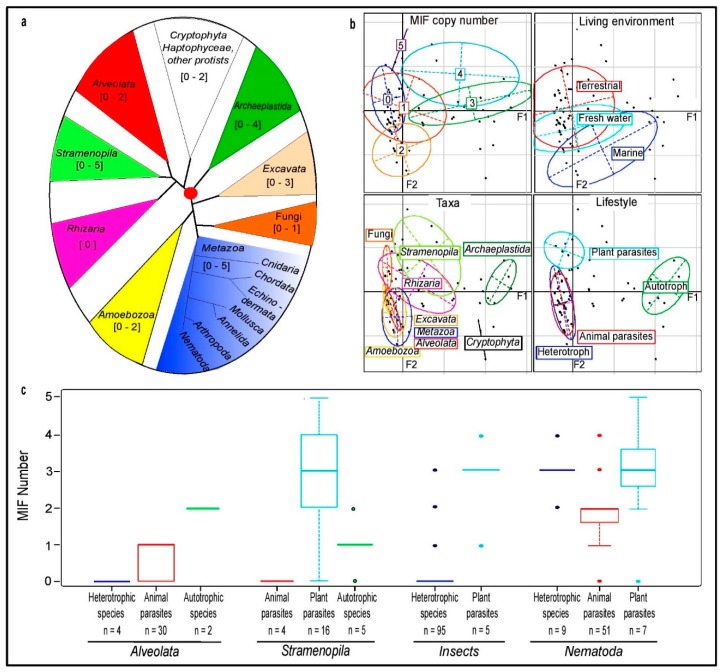
Analysis of MIF distribution across eukaryotic kingdoms. (**a**) Rooted phylogenetic tree of eukaryotic kingdoms according to Burki et al., 2014. The size of the triangle areas representing collapsed clades is not proportional to the number of taxa within each clade. The range of MIF sequence number identified in the respective species is indicated within square brackets. (**b**) Graphical representation of Multiple Correspondence Analysis (MCA) data showing, respectively, the distribution of MIF number, the environment, major taxa, and lifestyle. The confidence ellipses delimitate the centroid around each variable. The adjusted inertia of the F1 and F2 axes are 52.54% and 15.64% respectively. (**c**) Box plot analysis of MIF number according to the species lifestyle in *Alveolata* and *Stramenopila*, insects and nematodes (*n* = number of species). The vertical axis corresponds to the number of unique MIF protein sequences per species. Each box is delimited by the quartiles Q1 and Q3, whose variation Q3–Q1 corresponds to 50% of the distribution. The horizontal line in the center of the box represents the median value. The two ends of the vertical bars correspond to the values of the first and last percentiles (C1 and C99) and delimitate 98% of the sample distribution. Extreme values (outliers) are indicated by a dot (below 2% of the sample distribution).

Species possessing MIF protein sequences were detected in all main eukaryotic phyla with the exception of *Rhizaria* (Figure 1a). However, the absence of MIF from the entire taxon is uncertain as only five species have a sequenced genome (Supplementary Dataset 1).

To investigate a potential relation between the number of MIF sequences and taxonomic or ecological parameters, we removed from the analysis several basal eukaryotic taxonomic groups that were represented by only few species with available sequence data (i.e., *Haptophyceae*, *Apusozoa*, or *Parabasalia*), thus leaving a remaining total of 677 species out of the initial 803 species. The results from a Multiple Correspondence Analysis (MCA) [36] indicate that the species environment is poorly related to MIF number (Figure 1b). Although the median number of MIF per species is 1 for the marine and terrestrial environment, and 0 for the freshwater environment (Supplementary Figure S1a), a deviance analysis confirms that ‘environment’ is not a significant parameter for the prediction of MIF number (*P* > 0.05, generalized linear regression model). By contrast, MCA analysis shows that the distribution variation in MIF number is partly explained by both the parameters ‘taxa’ and ‘lifestyle’, that are strongly associated in fungi and *Archaeplastida.* Indeed, fungi comprise many plant-parasitic species and possess no, or one MIF at most, while *Archaeplastida*, exclusively represented by autotrophic species (i.e., plants, red and green algae), mostly possess three MIFs, which significantly differs from the other groups (*P* < 0.01, generalized linear regression model) (Supplementary Figure S1b).

In order to explore further the potential importance of ‘lifestyle’ as a parameter, while simultaneously reducing the confounding effect due to the phyla fungi and *Archaeplastida*, we focused on four other major phyla that show variation in MIF number and cover a substantial diversity of both free-living and parasitic species. These are the *Stramenopila*, *Alveolata*, and, among metazoans, the nematodes and insects (Figure 1c). Interestingly, while the global analysis, including the particular cases of fungi and *Archaeplastida*, suggested a reduced number of MIFs in plant-parasitic species (median value = 0, Supplementary Figure S1c), this phyla-specific study shows a higher number of MIF in plant parasites (Figure 1c). In *Stramenopila*, insects, and nematodes, the number of MIFs detected in organisms having a plant-parasitic lifestyle has a median value of 3 and is significantly higher than in the animal parasite groups (*P* < 0.01; Figure 1c). Taken together, and on the basis of detection of MIF sequences in public databases, these findings suggest (i) that free-living autotrophic species have a median number of 3 MIFs, (ii) that, with the exception of fungi, plant parasites also have a median number of 3 MIFs, while (iii) both heterotrophic and animal-parasitic species have a lower and variable number of MIFs.

### 3.2. MIF Phylogenetic Reconstruction across Kingdoms

MIF phylogenetic analyses were performed on a total of 720 high quality sequences presenting a full MIF domain and a minimum overlap of 85% with the rest of the aligned sequences. The details of specific sequences selected from the initial 803 species of plants, fungi, protists and animals for the phylogenetic analyses are provided in the Supplementary Dataset 1. Reconstructions were performed using the Bayesian Inference (BI) and the Maximum Likelihood (ML) methods. To avoid misinterpretation of poorly supported branches in the phylogenies, we used the Bayesian topology as a reference and reported the approximate likelihood-ratio test (aLRT) support values from the ML analysis together with the posterior probability (PP) values of the Bayesian analysis at corresponding branches.

**Figure 2:**
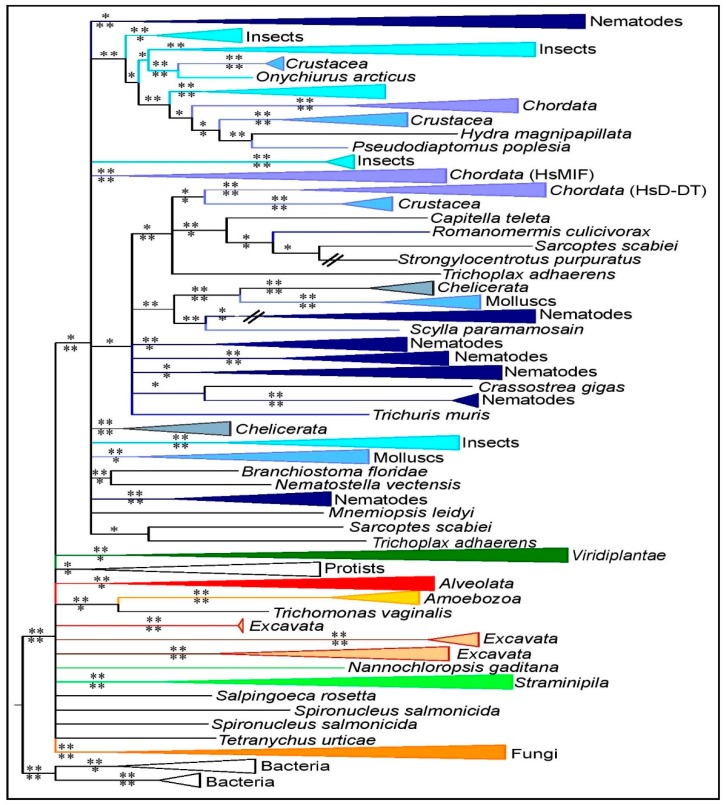
Overview of MIF phylogeny across eukaryotic kingdoms. Phylogenetic analysis of 720 MIF sequences based on alignments of 113 amino acid characters using the Bayesian Inference (BI) method and rooted using bacterial sequences (see Materials and Methods for details). Posterior probability (PP) support values are indicated above the branch for the BI and corresponding approximate likelihood-ratio test (aLRT) support values below the branch for the ML analysis. *: PP > 0.5 or aLRT > 0.7. **: PP > 0.7 or aLRT > 0.9. MIF clusters are colored according to the taxonomic phyla: metazoa (shades of blue), *Stramenopila* (light green), *Archaeplastida* (dark green), *Alveolata* (red), fungi (dark orange), *Excavata* (light orange), *Amoebozoa* (yellow), *Rhizari*a (pink), and *Cryptophyta*–*Haptophyceae*–*Picozoa* (white). Color codes of phyla are identical for all figures of the study.

A first observation is that the global phylogeny of MIF proteins does not correspond to the expected species tree (Figure 2). A second observation is that MIF sequences from *Viridiplantae*, *Alveolata*, *Stramenopila* and fungi form highly supported monophyletic groups (Figure 2), whereas the monophyly of other phyla is either not or only poorly supported in this analysis. Although the monophyly of metazoan MIFs is only moderately supported by the BI method, it is highly supported by the ML analysis (Figure 2), and it is consistent with observations from previous studies on metazoan MIFs [56]. A third observation is that the clear segregation of metazoan MIF and D-DT variants (Figure 2) previously reported in studies performed on a subset of taxa [56] is not supported in this cross-kingdom analysis, probably due to the poor resolution of most metazoan clades. Chordate MIFs form a monophyletic clade while MIF sequences from other metazoan phyla, such as nematodes, insects, or mollusks, do not form monophyletic groups in this analysis. Many metazoan phyla harbor one (or more) MIF(s) clustering with chordate D-DT variants, as well as one or more

MIF(s) that cannot be related to the chordate MIF or D-DT variants (Figure 2). This observation supports the idea that the metazoan MIFs originate from an ancient duplication event that occurred prior to metazoan diversification, approximately between 757 and 1.147 million years ago (MYA; according to TimeTree [51]), but the distinction between metazoan ‘MIF variant’ and ‘D-DT variant’ is not supported by our analysis.

In contrast to the situation in metazoans, the monophyly of plant MIFs is highly supported in our analysis (Figure 2, Supplementary Figure S2). However, the plant MIF tree itself does not match the expected species taxonomy. With the exception of some *Poaceae*, plant MIF phylogeny confirms that sequences cluster into three distinct groups that are related to the *Arabidopsis thaliana* MIFs, AtMDL1, AtMDL2, and AtMDL3 [15] (Supplementary Figure S2). This suggests two subsequent duplications in a common ancestor of the species that were considered.

In order to examine better the evolutionary history of MIFs in relation to possible constraints linked to a parasitic lifestyle, we then focused on phyla containing both free-living species, plant parasites and animal parasites, namely the fungi, *Stramenopila*, *Alveolata*, nematodes and insects. In the following phyla-specific studies, parts of the general tree (Figure 2) are expanded and detailed to show the precise phylogenetic relationships between subsets of the MIF sequences. As mentioned above, most fungal species do not possess typical MIF proteins, or only a single copy. The phylogeny of these few MIF sequences is consistent with the expected fungal species tree (Supplementary Figure S3).

### 3.3. MIFs in Stramenopila and Alveolata

**Figure 3:**
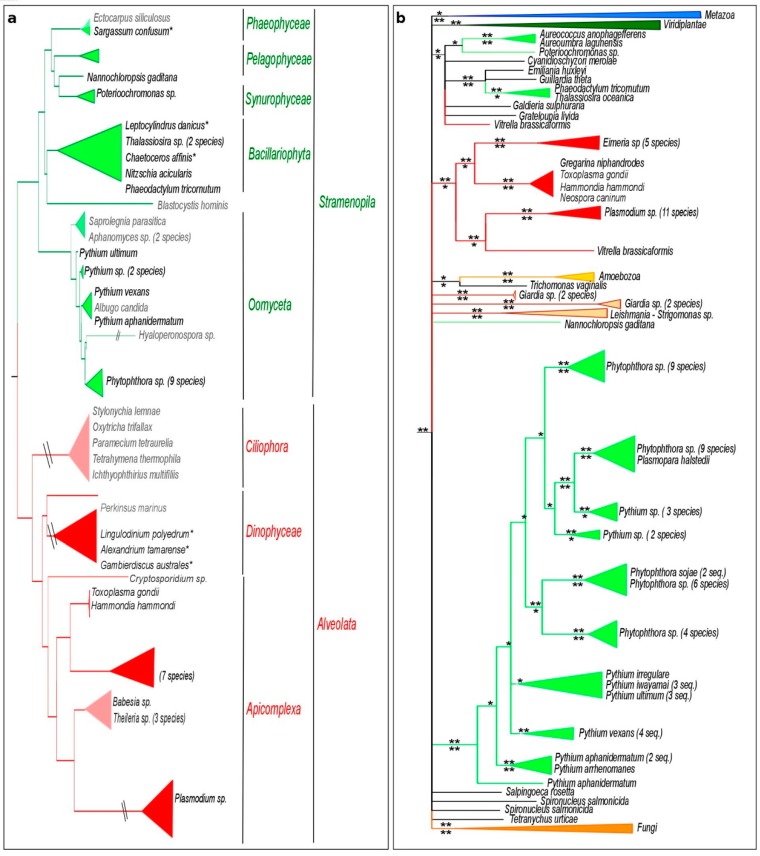
*Stramenopila* and *Alveolata* species tree and corresponding MIF phylogeny. (**a**) BI tree topology based on *Stramenopila* and *Alveolata* 18S rRNA sequences. (**b**) BI tree topology for MIF sequences of the same species. Species lacking an identified MIF sequence are shown in grey. The symbol // indicates that the respective branch does not follow the scale of the figure. Partial or atypical sequences are marked by a star in the species tree and are absent from the MIF tree. Colors of MIF clusters are given according to the taxonomy, as indicated in Figure 2. Posterior probability (PP) support values are indicated above the branch for the BI and corresponding aLRT support values below the branch for the ML analysis. *: PP > 0.5 or aLRT > 0.7. **: PP > 0.7 or aLRT > 0.9. The corresponding tree using the ML method is presented in the Supplementary Figure S4a.

The phylogenetic reconstruction of *Stramenopila* and *Alveolata* species, based on 18S rRNA analysis (Figure 3a) is consistent with the largely accepted species tree of these phyla [57]. Some phyla such as *Ciliophora* and some particular genera and species are missing MIF sequences, despite several genomes publicly available (Figure 3a). This observation may indicate that several independent gene loss events occurred in the *Stramenopila* and *Alveolata*. Species lacking MIF show a diversity of lifestyles. Some are free-living species such as *Ectocarpus siliculosus*, or *Paramecium tetraurelia*. Others are obligatory plant parasites, such as *Hyaloperonospora arabidopsidis*, or animal parasites such as *Saprolegnia parasitica*, and *Theileria sp*. Several *Stramenopila* and *Alveolata* species (marked by one star in the Figure 3a) appear to possess either a partial or an atypical MIF sequence with a MIF domain present within a longer unknown protein. Because of the uncertainty of these sequences, they were not included in the phylogenetic reconstruction analysis.

The MIF phylogenetic reconstruction overall matches the species tree, as the sequences from *Alveolata* and *Stramenopila* form two highly supported monophyletic clusters (Figure 3b). However, MIFs from one *Alveolata* species (*Vitrella brassicaformis*) and four *Stramenopila* species (*Aureococcus anophagefferens*, *Aureoumbra lagunensis*, *Phaeodactylum tricornutum*, and *Thalassiosira oceanica*) form a separate polyphyletic group. The main clustering of typical *Alveolata* or *Stramenopila* sequences is highly supported by both BI and ML methods (Figure 3b, Supplementary Figure S4). The positioning of MIFs from the five species *V. brassicaformis*, *A. anophagefferens*, *A. lagunensis*, *P. tricornutum*, and *T. oceanica* outside of the main *Stramenopila* and *Alveolata* clusters is also confirmed by both methods (Figure 3b, Supplementary Figure S4).

Within the *Stramenopila*, the oomycete sequences from the genera *Pythium* and *Phytophthora* do not form two separate monophyletic groups. Instead, they are interspersed multiple times. This suggests that duplication events occurred prior to the diversification of *Pythiales* and *Peronosporales*, which is estimated to have occurred approximately 80 MYA (TimeTree estimate [58]). Subsequent species-specific duplication events must have occurred more recently as both *Pythium* and *Phytophthora* species show multiple copies clustering together (Figure 3b).

For *Alveolata*, the MIF phylogeny broadly corresponds to the species tree, with the exception of MIFs from *Vitrella brassicaformis*. Two MIF proteins were identified from this dinoflagellate species and interestingly, one sequence clusters with other *Alveolata* sequences, while the second sequence groups with the ‘rogue’ MIF forming a separate group (Figure 3b, Supplementary Figure S4).

### 3.4. Nematode MIFs

**Figure 4:**
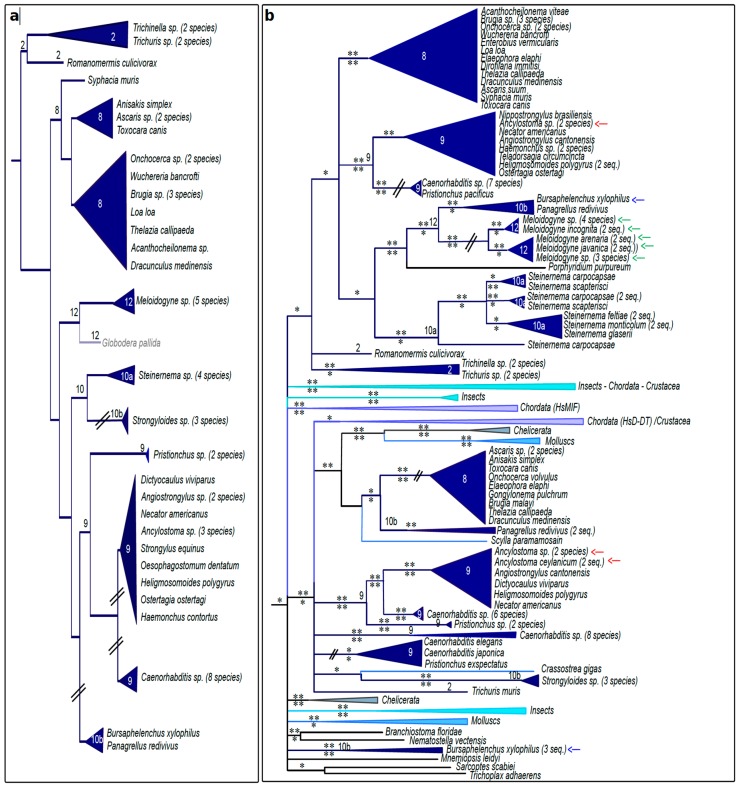
Nematode species tree and corresponding MIF phylogeny. (**a**) BI tree topology based on nematode 18S rRNA sequences. The nematode Clade numbers [59] are indicated at the corresponding branches for ease of results interpretation. The cyst nematode *Globodera pallida*, lacking any MIF protein sequence, is shown in grey. (**b**) BI tree topology for MIF sequences of the same species. Colors of MIF clusters are given according to the taxonomy, as indicated in Figure 2. MIF from three of the species commented in the text are indicated by arrows (*Ancylostoma* in red, *Meloidogyne* in green and *Bursaphelenchus* in blue). PP support values are indicated above the branch for the BI and corresponding aLRT support values below the branch for the ML analysis. *: PP > 0.5 or aLRT > 0.7. **: PP > 0.7 or aLRT > 0.9. The corresponding tree using the ML method is presented in Supplementary Figure S5a.

The nematode species tree obtained with the phylogenetic analysis of 18S rRNA genes (Figure 4a) is globally consistent with the currently accepted phylogeny of nematodes [59]. The only exceptions are the *Tylenchina*, which form three distinct groups, one with *Meloidogyne* and *Globodera*, one with *Steinernema* and *Strongyloides* and a last one with *Bursaphelenchus* and *Panagrellus*.

All nematode species with publicly available genome data present MIF proteins, with the noticeable exception of the cyst nematodes *Globodera pallida* and *Globodera rostochiensis* (Figure 4a). An extensive search of genomes and transcriptomes supported the absence of MIF sequences from these species and from their close relative in the *Heteroderidae*/*Hoplolaimidae* group. This finding and the presence of MIF in the root-knot nematodes (genus *Meloidogyne*) and in *Pratylenchidae*, belonging to the closest outgroups, suggests that MIF have been lost in a common ancestor of the *Heterodeidae*, *Rotylenchulidae* and *Hoplolaimidae*.

The phylogenetic reconstruction of MIF sequences from nematodes (Figure 4b) clearly differs from the 18S rRNA phylogenetic reconstruction (Figure 4a). As expected for many metazoans, some nematodes harbor two or more MIF proteins, apparently originating from an ancient duplication event occurring prior to nematode diversification, probably before the metazoan diversification (between 757 and 1147 MYA). As an example, the positions of the MIF proteins from the species *Ancylostoma* are indicated in Figure 4b. Interestingly, some phyla harbor several sets of MIF sequences apparently originating from more recent duplication events. For example, species of the genera *Meloidogyne* and *Steinernema* have two and three MIF variants, respectively, apparently resulting from duplication events that occurred after the separation of these genera from the other nematodes, and before the radiation and expansion of associated species. For *Meloidogyne*, the duplication probably arose before the diversification of the genus, about 78 MYA [60]. Unexpectedly, one MIF variant from the *Bursaphelenchus* species clusters with *Meloidogyne* MIFs, while a cluster of three sequences from the second variant is distant from other nematode MIFs.

### 3.5. Insect MIFs

**Figure 5:**
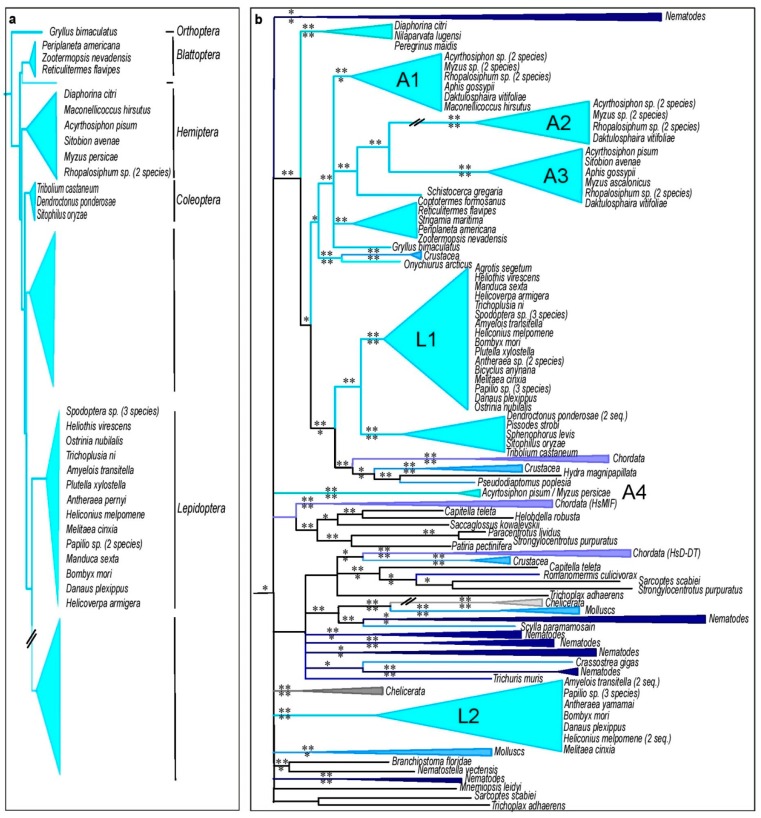
Insect species tree and corresponding MIF phylogeny. (**a**) BI tree topology based on insect 18S rRNA sequences. (**b**) BI tree topology for MIF sequences of the same species. The symbol // indicates that the respective branch does not follow the scale of the figure. Species and orders (*Diptera, Hymenoptera*, and *Phthiraptera*) lacking MIF sequences appear in grey. Colors of MIF clusters correspond to the species from which the MIF sequence originated. PP support values are indicated above the branch for the BI and corresponding aLRT support values below the branch for the ML analysis. *: PP > 0.5 or aLRT > 0.7. **: PP > 0.7 or aLRT > 0.9. The corresponding tree using the ML method is presented in Supplementary Figure S5b.

Insects show a diversity of MIF number, ranging from none to four. No MIF sequence were identified in the current databases for several clades such as *Hymenoptera, Diptera*, and *Phthiraptera* (Figure 5a), suggesting multiple independent losses of *MIF* genes in the common ancestors of each of these orders. The phylogenetic reconstruction of MIFs from insects clearly differs from the species tree and does not support a monophyletic relationship between insect MIF sequences and the MIF or D-DT variant from chordates (Figure 5b, Supplementary Figure S5b). The *Coleoptera*, *Blattodea*, and *Orthoptera* genera possess a single MIF sequence, while *Lepidoptera* species possess two variants belonging to two distantly related monophyletic groups (Indicated as L1 and L2 in the Figure 5b).

As previously shown, aphids possess multiple MIF sequences [18], and three of the aphid MIFs are closely related among each other (A1, A2, and A3 in the Figure 5b), while a fourth one (A4, Figure 5b) is strikingly different [18], supporting the suggestion that after the first duplication event expected to occur prior to metazoan diversification (between 757 and 1.147 MYA), subsequent serial duplications occurred before aphid diversification estimated around 90 MYA [61].

### 3.6. Conservation of Amino Acids and Motifs within MIF Sequences

To explore the possible conservation or diversification of particular amino acids and sequence motifs within MIF sequences from plant or animal parasites, we analyzed MIF sequences from five taxa, comprising *Alveolata* (parasites of animals), nematodes (plant and animal parasites considered separately), oomycetes (plant parasites), and aphids (plant parasites). For comparison, we also analyzed site selection in plant MIFs. Analyses were performed using five complementary site selection models (see Methods for details).

**Figure 6:**
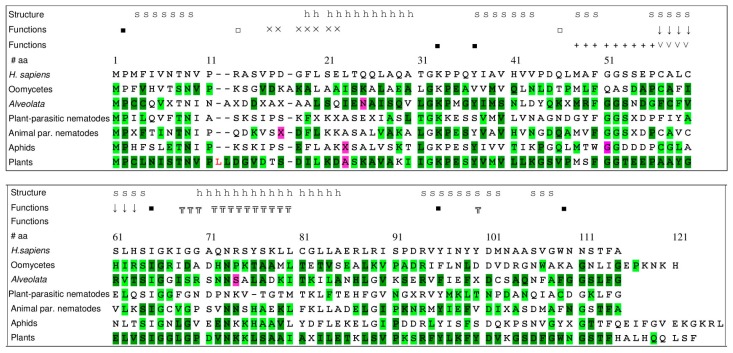
Conservation of amino acid sites and motifs in consensus MIF sequences. Alignment (amino acid one letter code) of human MIF sequences and consensus sequences of oomycetes, *Alveolata*, plant-parasitic nematodes, animal-parasitic nematodes, aphids and plants. Amino acid numbers are given above the human sequence. Hyphens (-) indicate a gap in the respective sequence. Consensus sequences, obtained using Jalview version 2.9, show the most frequent amino acids at each position, or an X when the frequency of the most prominent amino acid is below 40%. Sites under purifying or diversifying selection are colored as follow: pink = purifying selection predicted by 4 to 5 selection algorithms; light green = purifying selection predicted by two to three selection algorithms; dark green = purifying selection predicted by four to five selection algorithms. α helices and β sheets of human MIF are indicated by the letters ‘h’ and ‘s’, respectively. Amino acids shown to determine specific functions or activities in human MIF are specified by symbols above the sequence. They are involved in the tautomerase activity (■ symbol), oxidoreductase activity (CXXC motif; ˅ symbol), binding to the CXCR4 receptor (╦ symbol), binding to the CXCR2 receptor (pseudo ELR motif; □ symbol), interaction between MIF and the CXCR2 receptor (N like loop pattern; + symbol), nuclease activity (PE/DxxxxE motif; x symbol) and DNA damage response protein. (CxxCxxHx(n) zinc finger domain; ↓ symbol).

The conservation of plant MIFs [15] is confirmed here as 94 out of the 122 residues are predicted to be under purifying selection by four or five selection models (Figure 6). The other taxonomic groups considered (oomycetes, *Alveolata*, plant-parasitic nematodes, animal-parasitic nematodes, and aphids) include sequences from a lower number of species that are phylogenetically closer than the plant species. They show a number of strongly conserved amino acid residues varying from 31 to 75, and a comparatively low number of amino acid positions (six in total in the different taxonomic groups) are under positive (diversifying) selection. These positions are different in the various taxonomic groups and concern amino acids that are in part under purifying selection in other taxa. Interestingly, the residue corresponding to the leucine in position 23 in human MIF is predicted to be under positive selection in both plant and aphid sequences (Figure 6).

Remarkably, the MIF tautomerase active sites, encompassing residues Pro-2, Lys-33, Ile-65, Tyr-37, and Tyr-96, (symbol ■ in Figure 6) are globally conserved for most of the species, suggesting potential tautomerase activity of these MIF proteins. The presence of this enzymatic activity in bacterial MIF [62] and its conservation in most eukaryotic species support an ancestral origin of this function. Although a physiologic function of the tautomerase activity has not yet been identified in mammals, it is of note, that the Pro-2 residue has also been suggested to be involved in binding of mammalian MIF to the cognate receptor CD74 and the chemokine receptor CXCR4 [63].

Except for the tautomerase active sites, most residues or motifs known to be important for human MIF function are poorly conserved in the other groups. For example, the CXXC motif (Cys-57 and Cys-60) (˅ symbol in Figure 6) is only present in the consensus sequence of animal-parasitic nematodes. This site was previously described to be associated with the regulation of cellular redox homeostasis [64]; it is the active site of the MIF oxidoreductase (TPOR; thiol-protein oxidoreductase) activity. The limited presence of this motif might indicate that the regulation of redox homeostasis is probably not the primary ancestral MIF function.

The pseudo-(E)LR motif (Arg-12, Asp-45), which resembles the ELR motif of ELR+ CXC-type chemokines, was previously described to be important for an engagement of the chemokine receptor CXCR2 by MIF [65]. This motif is absent from the MIFs of the species we tested here, whereas the N-like loop motif, spanning residues 47–56 of human MIF, is partially conserved (e.g., compare LMAFGGSSEP in human MIF with PMSFGGTEE in plant MIF or LMTWGGDDDP in aphid MIF). The N-like loop motif is also involved in human MIF binding to the chemokine receptor CXCR4, but interaction of MIF with this receptor requires additional contributions from the residues RLR in position 87–89 [66]. Again, this extended N-like loop is only partially conserved in MIFs from the species studied herein.

A Tyr-100 residue in human MIF was recently identified as a solvent channel gating residue that dynamically interacts with and influences the CD74 activation site of MIF and thus is important for an allosteric mechanism of receptor stimulation [67]. This Tyr-100 residue is present in plant MIFs, but otherwise not conserved in the MIFs of the species tested here.

Finally, a nuclease activity was uncovered for human MIF, which has been suggested to involve the PD/ExxxxE motif (Pro-17 to Glu-22) [7]. This motif is absent from MIFs in other species, just like the zinc finger domain CxxCxxHx(n) (Cys-57 to His-63) commonly found in DNA damage response proteins, which seems also be required for nuclease activity [7].

## 4. Discussion

Our large-scale data mining effort involving over 800 species confirmed that MIF proteins are present in all eukaryotic kingdoms and suggested that the number of MIF varies from 0 to 5 in the taxa analyzed. This first broad survey of MIF presence across species of plants, fungi, protists, and animals may underestimate the total number of MIF proteins in some taxa. Firstly, we focused on typical MIF proteins mainly composed of a MIF domain. During our search, MIF domains have also been found in association with other domains in complex multidomain proteins, in particular in fungi and *Alveolata*. None of these complex proteins have been reported in published MIF studies so far and they deserve future dedicated evolutionary and functional investigations. It is therefore likely that a future study on MIF domain-containing proteins would provide different results. Secondly, MIF number may also be underestimated in some taxa because the sequence databases (genomes, transcriptomes, etc.) are incomplete and present varying degrees of depth, quality, and curation. For example, the apparent absence of MIF in *Rhizaria* is likely due to the lack of sequence data (only 5 genomes sequenced). In contrast, the low number of typical MIF sequences identified in fungi cannot be related to a poor database quality as the fungal kingdom is one of the most extensively studied in terms of high-quality genomes and transcriptomes. Similarly, important sequencing efforts have been made on plants and on parasitic species (whether animal or plant parasites) belonging to various taxa, because of their medical, veterinary or agricultural importance. It is therefore likely that underestimation of MIF number occurred mostly in free-living heterotrophic taxa such as echinoderms, annelids or myriapods that are not the focus of the present study.

Important observations from this analysis are that, (i) most of the *Archaeplastida*, represented by free-living autotrophic species, harbor three MIFs, (ii) with the exception of fungi, plant parasites belonging to phylogenetically distant groups such as *Stramenopila*, insects or nematodes, also harbor a median number of three MIFs, while (iii) both free-living heterotrophic species and animal parasite species harbor a lower and/or variable number of MIFs. This similarity of median MIF number between plants and plant-parasitic species raises the question of putative functional constraints on MIF numbers in plant parasites.

Previous phylogenetic studies on MIF/D-DT (D-DT now also often referred to as MIF-2 [68]) in metazoans were either restricted to specific taxonomic groups such as insects [18], arthropods [69], or mollusks [70], or were focused on the relation between sequences from a single species and known sequences from vertebrates [14]. Several of these phylogenies supported a classification of MIF and D-DT variants from metazoans [13,71]. The present extended cross-kingdom analysis shows that MIF sequences from most metazoan phyla, such as nematodes, insects, or mollusks, do not form monophyletic groups. While a number of MIF sequences from metazoans such as crustacean, mollusks, echinoderms, and nematodes form a highly supported cluster with the chordate D-DT sequences, most other sequences including sequences from metazoans, plants, and protists form independent early-branching clusters that cannot be related either to the chordate MIF or chordate D-DT. One of the reasons for this is that most of the basal nodes in the phylogeny are not resolved and are represented as a polytomy.

**Figure 7:**
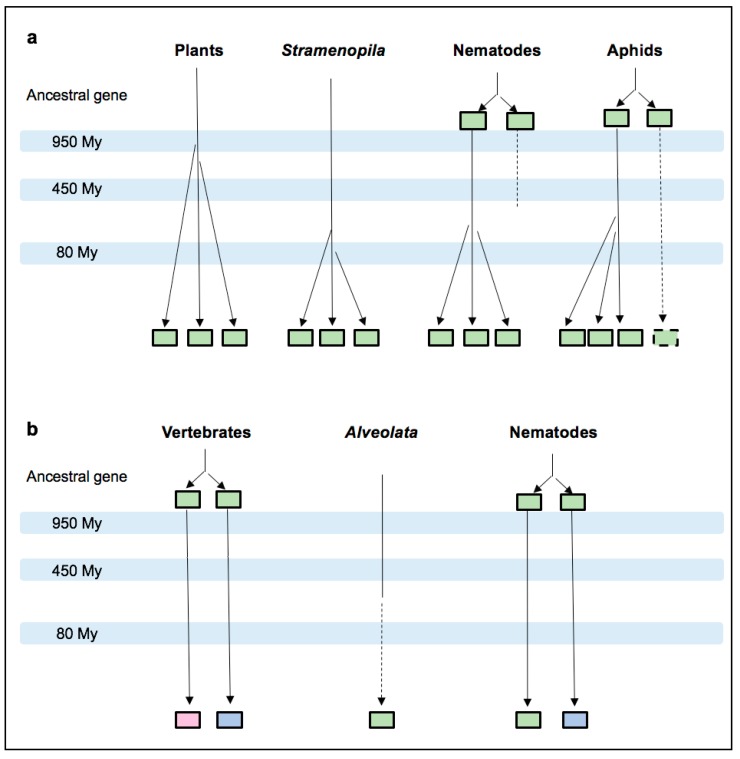
Evolutionary model showing MIF duplication events in phyla from selected eukaryotic kingdoms. The hypothesized duplication events are indicated on simplified lineage trees reconciling the median MIF number analysis and MIF phylogeny, for plants and plant parasites belonging to protists or metazoans (**a**), and for vertebrate and vertebrate parasites belonging to protists or metazoans (**b**). Light green boxes represent MIF members that cannot be related to the chordate MIF or D-DT variants in our study. Light pink and light blue boxes represent sequences associated with the chordate MIF and D-DT, respectively. Dashed lines indicate the apparent loss of one MIF member. The approximate time scale is based on median estimate from TimeTree. For example, 950 MYA represents the period between 757 and 1147 MYA.

Similar to variations in MIF number, the MIF evolutionary history appears different according to phyla. In fungi, the phylogenetic MIF tree broadly follows the species tree. This observation, together with the low number (0–1) of MIF detected, suggest that MIF is not functionally essential and therefore subject to selection in this taxonomic group. By contrast, as previously reported, both the number and the phylogenetic tree of plant MIFs (MDLs) suggest that they are functionally important and originate from two serial duplication events [15]. This expected functional importance is further supported here by the observation of a strong purifying selection acting on 94 out of 122 residues of MDLs. While ancient duplications resulted in the existence of the three plant MDLs, the presence of three or more MIFs in plant parasites such as oomycetes (protists), nematodes or aphids (insects) seems to result from different evolutionary events involving recent clade-specific duplications (Figure 7a). By contrast, the phylogeny of MIF in animal parasites such as *Alveolata* (protists) or nematodes does not support recent duplication events (Figure 7b).

Altogether, our results support the idea that the occurrence of three MIF copies is functionally important for both plants and plant parasites, regardless of their taxonomic status. To date, studies on MIF functions in plants and plant parasites are extremely scarce. Plant MDLs have not been functionally studied yet. However, an in depth in silico analysis suggested that two *Arabidopsis thaliana* *MDL* genes (*AtMDL1* and *2*) are constitutively expressed and share an overlapping set of co-expressed genes [15]. The third gene (*AtMDL3*) exhibits stress-inducible transcript accumulation and is co-expressed with a number of genes implicated in plant immunity [15]. To our knowledge, to date a single functional study on MIF from plant parasites showed that aphid MIF secreted during feeding can interfere with plant immune responses [16]. However, the present comparison of MIF sequences from plants, plant parasites or animal parasites, did not identify particular motifs that are specific for plant parasites since none of the 30 to 60 amino acids found to be under negative selection in the three groups of plant parasites (oomycetes, nematodes, aphids) appear to be shared exclusively by plant parasites.

Also, with the exception of the tautomerase active sites, most sites known to be functionally important in human MIF are not conserved. This situation supports an ancient origin of MIF tautomerase function, but does not allow prediction of other potential activities of these MIF proteins. Therefore, future functional studies should help deciphering MIF multifunctional activities in the various kingdoms.

## 5. Conclusions

This extensive analysis of MIF sequences from plant, fungi, protist and metazoan kingdoms shows that the evolutionary history of eukaryotic MIF sequences is complex, and suggests that both ancestral duplications, multiple gene losses and recent clade-specific reduplications occurred. Although the number of MIF variants per species is variable, ranging from none to five in the taxa analyzed, factors underlying the variation in MIF number remain unclear for most kingdoms, clades or species. Of note, the single general observation is that both plants (free-living autotrophic species) and plant parasites harbor a median number of three MIFs, while heterotrophic and animal-parasitic species harbor a lower and variable MIF number. Maybe even more surprising is that MIFs seem to be essential and highly conserved, with many sites under purifying selection in some kingdoms (e.g., plants), while in other kingdoms the protein appears more dispensable (e.g., in fungi) or present in several diverged variants (e.g., in insects and nematodes). This suggests potential neofunctionalizations apart from the ancestral and possibly conserved tautomerase activity. Future comparative functional studies across kingdoms should bring insights into functions and function-associated sites of these evolutionarily and functionally complex proteins and may ultimately help to pinpoint currently unrecognized molecular target sites that could be applicable in MIF-based therapeutic or plant protection strategies.

## Supplementary Materials

The following are available online at https://www.mdpi.com/2073-4425/10/10/740/s1, Table S1: Comprehensive list of species searched for MIF protein sequences, Table S2: Species and accession numbers of MIF sequences used as queries for the BLAST searches, Figure S1: MIF copy number as a function of taxa, environment, and lifestyle, Figure S2: Plant MIF phylogeny, Figure S3: MIF phylogeny of fungal species, Figure S4: Maximum likelihood analyses of MIF sequences from Stramenopila and Alveolata, Figure S5: Maximum likelihood phylogeny of MIF sequences from nematodes and insects. The dataset supporting the phylogenetic analyses is available in the TreeBase repository (http://purl.org/phylo/treebase/phylows/study/TB2:S21992?x-access-code=4a0cbb6434be939b700c0fee1c363e8d&format=html).
