# Supplementary material for: Cross-Kingdom Analysis of Diversity, Evolutionary History, and Site Selection within the Eukaryotic Macrophage Migration Inhibitory Factor Superfamily
Source: Genes (Basel). 2019 Sep 24;10(10):740. doi: 10.3390/genes10100740 (PMC6826473; doi:10.3390/genes10100740)
Supplement: Supplementary file 1 [file genes-10-00740-s001.zip › Michelet suppl/Michelet et al Supplementary figures.pdf]

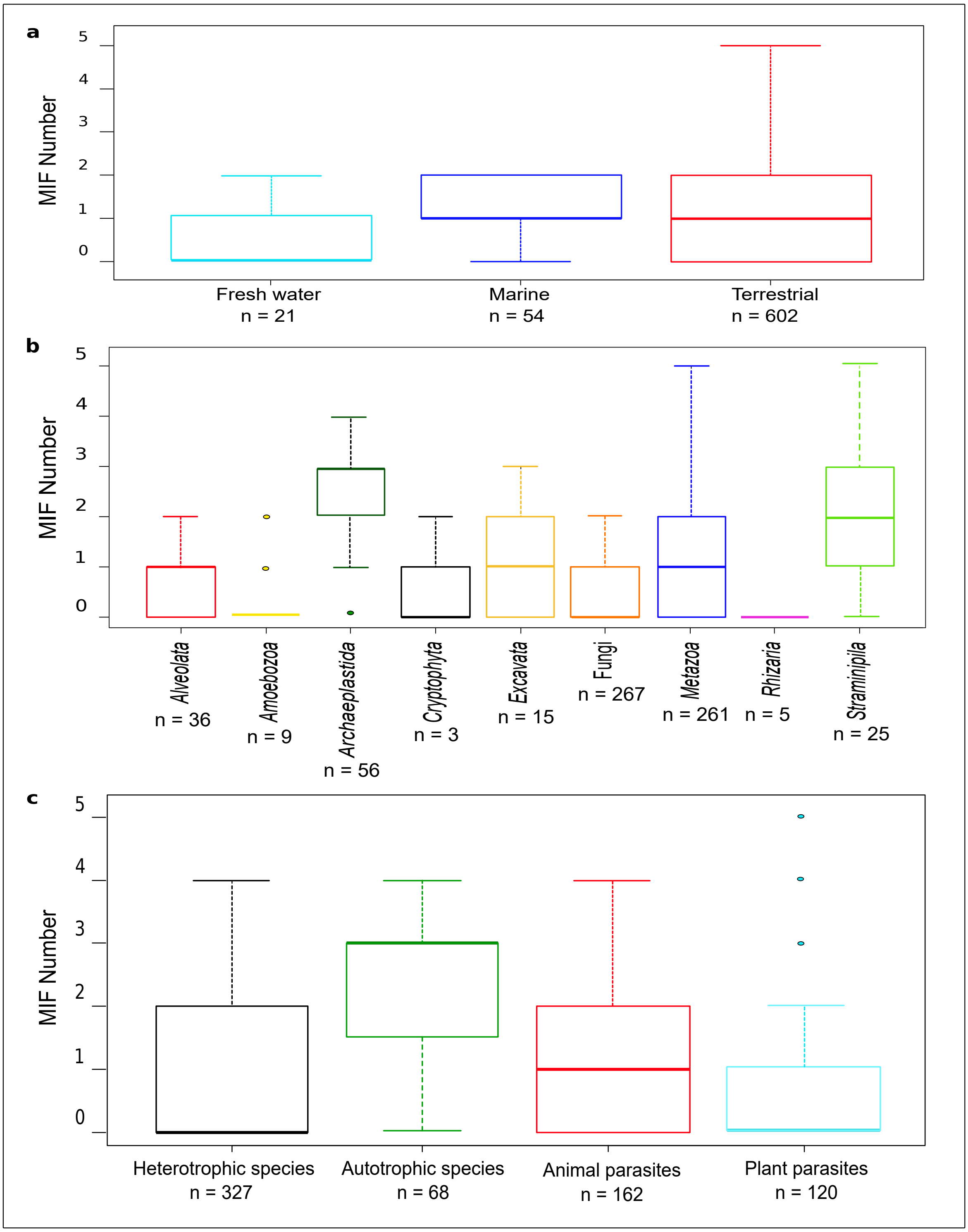

Figure S1: MIF copy number as a function of taxa, environment, and lifestyle. Box plot of the differences in MIF copy number according to the environment (**a**), the taxon (**b**) and the lifestyle (**c**) of species. Each box is delimited by the quartiles Q1 and Q3, whose variation Q3-Q1 corresponds to 50% of the species. The horizontal line in the centre of the box represents the median value. The two ends of the vertical bars correspond to the values of the first and last percentiles (C1 and C99) and delimit 98 % of the sample distribution. Extreme values (outliers) are indicated by a dot (below 2% of the sample distribution).

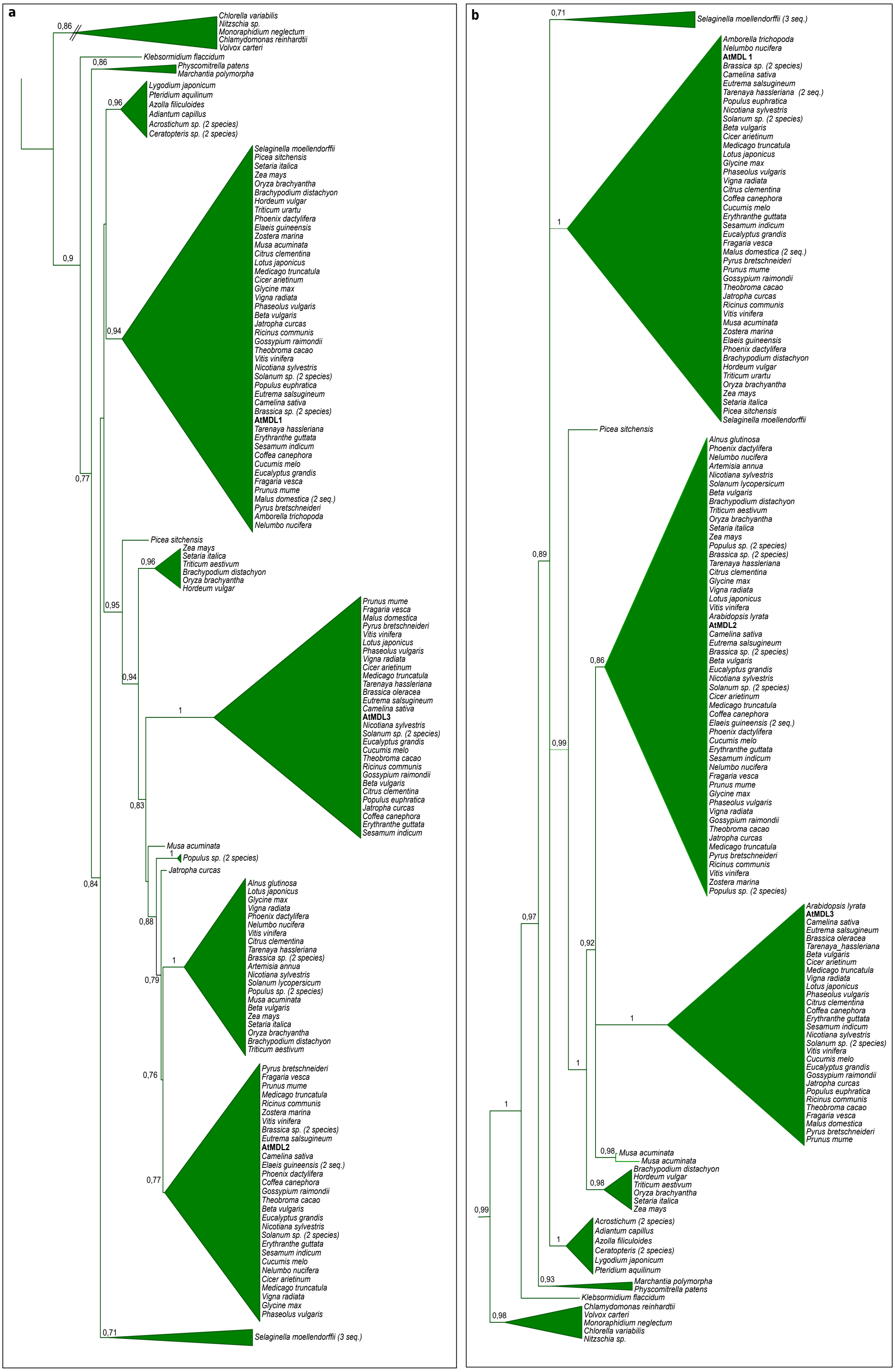
a
0,86
Chlorella variabilis
Nitzschia sp.
Monoraphidium neglectum
Chlamydomonas reinhardtii
Volvox carteri
Klebsormidium flaccidum
0,86
Physcomitrella patens
Marchantia polymorpha
0,96
Lygodium japonicum
Pteridium aquilinum
Azolla filiculoides
Adiantum capillus
Acrostichum sp. (2 species)
Ceratopteris sp. (2 species)
Selaginella moellendorffii
Picea sitchensis
Setaria italica
Zea mays
Oryza brachyantha
Brachypodium distachyon
Hordeum vulgar
Triticum urartu
Phoenix dactylifera
Elaeis guineensis
Zostera marina
Musa acuminata
Citrus clementina
Lotus japonicus
Medicago truncatula
Cicer arietinum
Glycine max
Vigna radiata
Phaseolus vulgaris
Beta vulgaris
Jatropha curcas
Ricinus communis
Gossypium raimondii
Theobroma cacao
Vitis vinifera
Nicotiana sylvestris
Solanum sp. (2 species)
Populus euphratica
Eutrema salsugineum
Camelina sativa
Brassica sp. (2 species)
AtMDL1
Tarenaya hassleriana
Erythranthe guttata
Sesamum indicum
Coffea canephora
Cucumis melo
Eucalyptus grandis
Fragaria vesca
Prunus mume
Malus domestica (2 seq.)
Pyrus bretschneideri
Amborella trichopoda
Nelumbo nucifera
0,9
0,94
0,77
Picea sitchensis
0,96
Zea mays
Setaria italica
Triticum aestivum
Brachypodium distachyon
Oryza brachyantha
Hordeum vulgar
0,95
Prunus mume
Fragaria vesca
Malus domestica
Pyrus bretschneideri
Vitis vinifera
Lotus japonicus
Phaseolus vulgaris
Vigna radiata
Cicer arietinum
Medicago truncatula
Tarenaya hassleriana
Brassica oleracea
Eutrema salsugineum
Camelina sativa
AtMDL3
Nicotiana sylvestris
Solanum sp. (2 species)
Eucalyptus grandis
Cucumis melo
Theobroma cacao
Ricinus communis
Gossypium raimondii
Beta vulgaris
Citrus clementina
Populus euphratica
Jatropha curcas
Coffea canephora
Erythranthe guttata
Sesamum indicum
0,94
1
0,83
Musa acuminata
1
Populus sp. (2 species)
Jatropha curcas
0,84
0,88
Alnus glutinosa
Lotus japonicus
Glycine max
Vigna radiata
Phoenix dactylifera
Nelumbo nucifera
Vitis vinifera
Citrus clementina
Tarenaya hassleriana
Brassica sp. (2 species)
Artemisia annua
Nicotiana sylvestris
Solanum lycopersicum
Populus sp. (2 species)
Musa acuminata
Beta vulgaris
Zea mays
Setaria italica
Oryza brachyantha
Brachypodium distachyon
Triticum aestivum
1
0,79
0,76
Pyrus bretschneideri
Fragaria vesca
Prunus mume
Medicago truncatula
Ricinus communis
Zostera marina
Vitis vinifera
Brassica sp. (2 species)
Eutrema salsugineum
AtMDL2
Camelina sativa
Elaeis guineensis (2 seq.)
Phoenix dactylifera
Coffea canephora
Gossypium raimondii
Theobroma cacao
Beta vulgaris
Eucalyptus grandis
Nicotiana sylvestris
Solanum sp. (2 species)
Erythranthe guttata
Sesamum indicum
Cucumis melo
Nelumbo nucifera
Cicer arietinum
Medicago truncatula
Vigna radiata
Glycine max
Phaseolus vulgaris
0,77
0,71
Selaginella moellendorffii (3 seq.)
b
0,71
Selaginella moellendorffii (3 seq.)
Amborella trichopoda
Nelumbo nucifera
AtMDL 1
Brassica sp. (2 species)
Camelina sativa
Eutrema salsugineum
Tarenaya hassleriana (2 seq.)
Populus euphratica
Nicotiana sylvestris
Solanum sp. (2 species)
Beta vulgaris
Cicer arietinum
Medicago truncatula
Lotus japonicus
Glycine max
Phaseolus vulgaris
Vigna radiata
Citrus clementina
Coffea canephora
Cucumis melo
Erythranthe guttata
Sesamum indicum
Eucalyptus grandis
Fragaria vesca
Malus domestica (2 seq.)
Pyrus bretschneideri
Prunus mume
Gossypium raimondii
Theobroma cacao
Jatropha curcas
Ricinus communis
Vitis vinifera
Musa acuminata
Zostera marina
Elaeis guineensis
Phoenix dactylifera
Brachypodium distachyon
Hordeum vulgar
Triticum urartu
Oryza brachyantha
Zea mays
Setaria italica
Picea sitchensis
Selaginella moellendorffii
1
Picea sitchensis
Alnus glutinosa
Phoenix dactylifera
Nelumbo nucifera
Artemisia annua
Nicotiana sylvestris
Solanum lycopersicum
Beta vulgaris
Brachypodium distachyon
Triticum aestivum
Oryza brachyantha
Setaria italica
Zea mays
Populus sp. (2 species)
Brassica sp. (2 species)
Tarenaya hassleriana
Citrus clementina
Glycine max
Vigna radiata
Lotus japonicus
Vitis vinifera
Arabidopsis lyrata
AtMDL2
Camelina sativa
Eutrema salsugineum
Brassica sp. (2 species)
Beta vulgaris
Eucalyptus grandis
Nicotiana sylvestris
Solanum sp. (2 species)
Cicer arietinum
Medicago truncatula
Coffea canephora
Elaeis guineensis (2 seq.)
Phoenix dactylifera
Cucumis melo
Erythranthe guttata
Sesamum indicum
Nelumbo nucifera
Fragaria vesca
Prunus mume
Glycine max
Phaseolus vulgaris
Vigna radiata
Gossypium raimondii
Theobroma cacao
Jatropha curcas
Medicago truncatula
Pyrus bretschneideri
Ricinus communis
Vitis vinifera
Zostera marina
Populus sp. (2 species)
0,89
0,86
0,99
0,97
0,92
Arabidopsis lyrata
AtMDL3
Camelina sativa
Eutrema salsugineum
Brassica oleracea
Tarenaya hassleriana
Beta vulgaris
Cicer arietinum
Medicago truncatula
Vigna radiata
Lotus japonicus
Phaseolus vulgaris
Citrus clementina
Coffea canephora
Erythranthe guttata
Sesamum indicum
Nicotiana sylvestris
Solanum sp. (2 species)
Vitis vinifera
Cucumis melo
Eucalyptus grandis
Gossypium raimondii
Jatropha curcas
Populus euphratica
Ricinus communis
Theobroma cacao
Fragaria vesca
Malus domestica
Pyrus bretschneideri
Prunus mume
1
1
1
0,98
Musa acuminata
Musa acuminata
0,98
Brachypodium distachyon
Hordeum vulgar
Triticum aestivum
Oryza brachyantha
Setaria italica
Zea mays
0,99
Acrostichum (2 species)
Adiantum capillus
Azolla filiculoides
Ceratopteris (2 species)
Lygodium japonicum
Pteridium aquilinum
1
0,93
Marchantia polymorpha
Physcomitrella patens
Klebsormidium flaccidum
0,98
Chlamydomonas reinhardtii
Volvox carteri
Monoraphidium neglectum
Chlorella variabilis
Nitzschia sp.

Figure S2: Plant MIF phylogeny. Phylogenetic analyses of plant MIF sequences using the Bayesian Inference (BI) method (**a**) and the maximum likelihood (ML) method (**b**). The *Arabidopsis thaliana* MIF sequences (AtMDL) are shown in bold. Statistical confidence values are indicated next to each branch, when they were superior to 0.5 and 0.7, according to the BI method and the approximate likelihood-ratio test (aLRT) with maximum likelihood analyses, respectively.

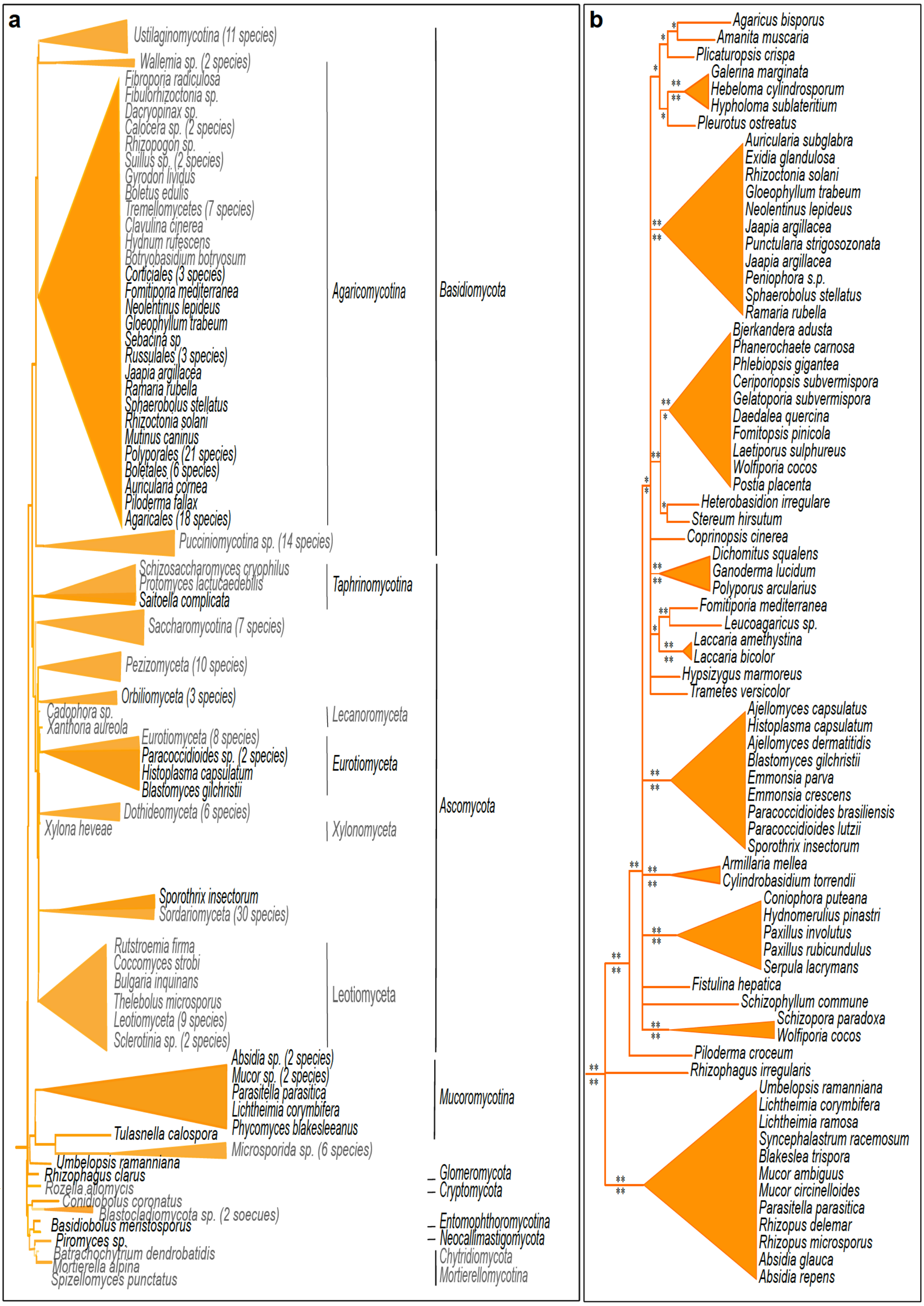
a
Ustilaginomycotina (11 species)
Wallemia sp. (2 species)
Fibroporia radiculosa
Fibulorhizoctonia sp.
Dacryopinax sp.
Calocera sp. (2 species)
Rhizopogon sp.
Suillus sp. (2 species)
Gyrodon lividus
Boletus edulis
Tremellomycetes (7 species)
Clavulina cinerea
Hydnum rufescens
Botryobasidium botryosum
Corticiales (3 species)
Fomitiporia mediterranea
Neolentinus lepideus
Gloeophyllum trabeum
Sebacina sp
Russulales (3 species)
Jaapia argillacea
Ramaria rubella
Sphaerobolus stellatus
Rhizoctonia solani
Mutinus caninus
Polyporales (21 species)
Boletales (6 species)
Auricularia cornea
Piloderma fallax
Agaricales (18 species)
Pucciniomycotina sp. (14 species)
Agaricomycotina
Basidiomycota
Schizosaccharomyces cryophilus
Protomyces lactucaedebilis
Saitoella complicata
Taphrinomycotina
Saccharomycotina (7 species)
Pezizomyceta (10 species)
Orbiliomyceta (3 species)
Cadophora sp.
Xanthoria aureola
Lecanoromyceta
Eurotiomyceta (8 species)
Paracoccidioides sp. (2 species)
Histoplasma capsulatum
Blastomyces gilchristii
Eurotiomyceta
Dothideomyceta (6 species)
Xylona heveae
Xylonomyceta
Ascomycota
Sporothrix insectorum
Sordariomyceta (30 species)
Rutstroemia firma
Coccomyces strobi
Bulgaria inquinans
Thelebolus microsporus
Leotiomyceta (9 species)
Sclerotinia sp. (2 species)
Leotiomyceta
Absidia sp. (2 species)
Mucor sp. (2 species)
Parasitella parasitica
Lichtheimia corymbifera
Phycomyces blakesleeanus
Mucoromycotina
Tulasnella calospora
Microsporida sp. (6 species)
Umbelopsis ramanniana
Rhizophagus clarus
Rozella allomycis
Conidiobolus coronatus
Blastocladiomycota sp. (2 soecues)
Basidiobolus meristosporus
Piromyces sp.
Batrachochytrium dendrobatidis
Mortierella alpina
Spizellomyces punctatus
Glomeromycota
Cryptomycota
Entomophthoromycotina
Neocallimastigomycota
Chytridiomycota
Mortierellomycotina
b
Agaricus bisporus
Amanita muscaria
Plicaturopsis crispa
Galerina marginata
Hebeloma cylindrosporum
Hypholoma sublateritium
Pleurotus ostreatus
Auricularia subglabra
Exidia glandulosa
Rhizoctonia solani
Gloeophyllum trabeum
Neolentinus lepideus
Jaapia argillacea
Punctularia strigosozonata
Jaapia argillacea
Peniophora s.p.
Sphaerobolus stellatus
Ramaria rubella
Bjerkandera adusta
Phanerochaete carnosa
Phlebiopsis gigantea
Ceriporiopsis subvermispora
Gelatoporia subvermispora
Daedalea quercina
Fomitopsis pinicola
Laetiporus sulphureus
Wolfiporia cocos
Postia placenta
Heterobasidion irregulare
Stereum hirsutum
Coprinopsis cinerea
Dichomitus squalens
Ganoderma lucidum
Polyporus arcularius
Fomitiporia mediterranea
Leucoagaricus sp.
Laccaria amethystina
Laccaria bicolor
Hypsizygus marmoreus
Trametes versicolor
Ajellomyces capsulatus
Histoplasma capsulatum
Ajellomyces dermatitidis
Blastomyces gilchristii
Emmonsia parva
Emmonsia crescens
Paracoccidioides brasiliensis
Paracoccidioides lutzii
Sporothrix insectorum
Armillaria mellea
Cylindrobasidium torrendii
Coniophora puteana
Hydnomerulius pinastri
Paxillus involutus
Paxillus rubicundulus
Serpula lacrymans
Fistulina hepatica
Schizophyllum commune
Schizopora paradoxa
Wolfiporia cocos
Piloderma croceum
Rhizophagus irregularis
Umbelopsis ramanniana
Lichtheimia corymbifera
Lichtheimia ramosa
Syncephalastrum racemosum
Blakeslea trispora
Mucor ambiguus
Mucor circinelloides
Parasitella parasitica
Rhizopus delemar
Rhizopus microsporus
Absidia glauca
Absidia repens

Figure S3: MIF phylogeny of fungal species. Results of Bayesian Inference (BI) analyses based on fungal 18S RNA sequences (**a**) and corresponding MIF sequences (**b**). Statistical confidence values are indicated next to each branch, when they were superior to 0.5 and 0.7, according to the BI method and the approximate likelihood-ratio test (aLRT) with maximum likelihood analyses, respectively. The species tree is consistent with the currently accepted phylogeny of fungi [75]. Species lacking MIF sequences are shown in grey.

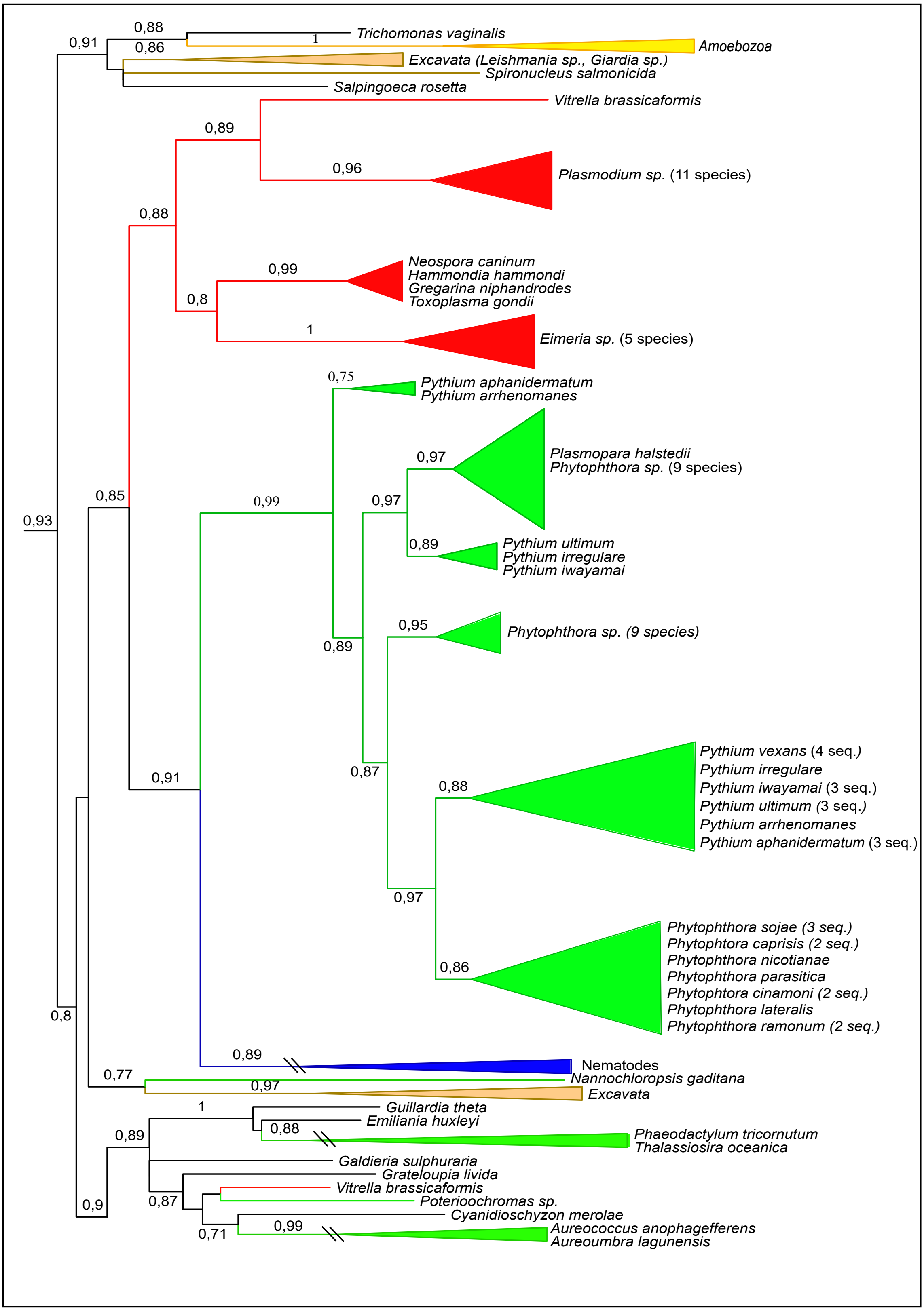

Figure S4: Maximum likelihood analyses of MIF sequences from *Stramenopila* and *Alveolata*. Results of maximum likelihood (ML) analysis of MIF sequences from *Stramenopila* and *Alveolata*. Statistical confidence values are indicated next to each branch when superior to 0.7 according to the approximate likelihood-ratio test (aLRT).

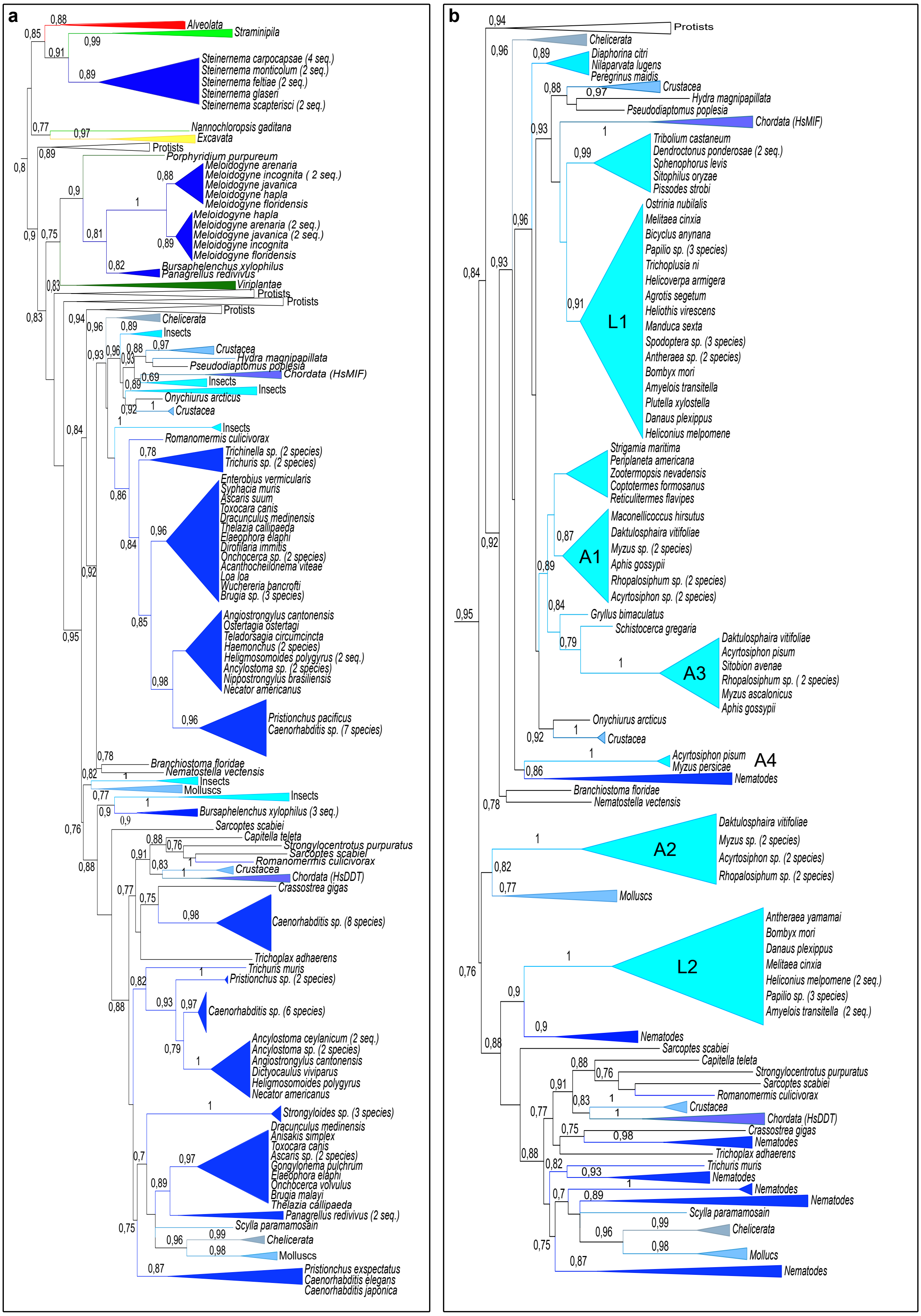

Figure S5: Maximum likelihood phylogeny of MIF sequences from nematodes and insects. Results of maximum likelihood (ML) analyses of MIF sequences from nematodes (**a**) and

insects (**b**). Statistical confidence values are indicated next to each branch when superior to 0.7 according to the approximate likelihood-ratio test (aLRT).
