# Supplementary material for: Cross-Kingdom Analysis of Diversity, Evolutionary History, and Site Selection within the Eukaryotic Macrophage Migration Inhibitory Factor Superfamily
Source: Genes (Basel). 2019 Sep 24;10(10):740. doi: 10.3390/genes10100740 (PMC6826473; doi:10.3390/genes10100740)
Supplement: Supplementary file 1 [file genes-10-00740-s001.zip › Michelet suppl/Table S1.pdf]

Table S1 : Comprehensive list of species searched for MIF protein sequences. The table shows the number of typical MIF sequences identified, the general environment (terrestrial, freshwater or marine), the life-style (free-living autotrophs, heterotrophs, and animal- or plant-parasites), as well as a short description (e.g. brown algae, fungi parasite, etc.). The presence/absence of a sequenced genome at the time of the search is indicated. The MIF sequences selected for the phylogenic reconstructions are indicated. Finally, the number of ESTs available at the time of the study are shown for species that did not possess a sequenced genome.

Phyla	Species	MIF number	Environment	Life-style	Description	Sequenced Genome	Selected for phylogeny reconstruction	ESTs (Number)
**Cryptophyta**								
	Chroomonas mesostigmatica	0	marine	autotrophic species	algaes	present	absent	
	Cryptomonas paramecium	0	marine	autotrophic species	algaes	present	absent	
	Guillardia theta	2	marine	autotrophic species	algaes	present	yes	
**Apusozoa**								
	Thecamonas trahens	0	marine	heterorophic species	free living, biflagellate	present	absent	
**Parabasalia**								
	Trichomonas vaginalis	1	terrestrial	animal parasites	causes uro genital trichomonoze	present	yes	
**Haptophyta**								
	Emiliania huxleyi	1	marine	autotrophic species	algaes, phytoplankton, causes blooms	present	yes	
**Rhizaria --> Cercomonads**								
	Bigelowiella natans	0	marine	autotrophic species	model organisms, algaes	present	absent	
**Rhizaria --> Cercozoa**								
	Lotharella oceanica	0	marine	autotrophic species	chlorarachniophyte algaes, plankton	present	absent	
	Paulinella chromatophora	0	freshwater	heterorophic species	amoeba - free living - photosynthetic	present	absent	
	Plasmodiophora brassicae	0	terrestrial	plant parasites	causes clubroot	present	absent	
**Rhizaria --> Foraminifera**								
	Reticulomyxa filosa	0	freshwater	heterorophic species	foraminifers	present	absent	
**Excavates --> Diplomonads**								
	Giardia intestinalis	2	terrestrial	animal parasites	causes giardiasis, flagellated parasite	present	yes	
	Giardia lamblia	2	terrestrial	animal parasites	causes giardiasis, flagellated parasite	present	yes	
	Spironucleus salmonicida	2	terrestrial	animal parasites	fish parasite, systemic infection in salmonid fish	present	yes	
**Excavates --> Kinetoplastids**								
	Leishmania braziliensis	1	terrestrial	animal parasites	causes leishmaniasis	present	no	
	Leishmania donovani	2	terrestrial	animal parasites	causes leishmaniasis	present	yes	
	Leishmania infantum	2	terrestrial	animal parasites	causes leishmaniasis	present	yes	
	Leishmania major	2	terrestrial	animal parasites	causes leishmaniasis	present	yes	
	Leishmania mexicana	2	terrestrial	animal parasites	causes leishmaniasis	present	yes	
	Leishmania panamensis	1	terrestrial	animal parasites	causes leishmaniasis	present	no	
	Phytomonas sp	0	terrestrial	plant parasites	conveyed by bugs	present	absent	
	Strigomonas culicis	1	terrestrial	animal parasites	infects and colonizes the Aedes aegypti	present	yes	
	Trypanosoma brucei	0	terrestrial	animal parasites	causes trypanosomiasis, flagellate parasite	present	absent	
	Trypanosoma cruzi	0	terrestrial	animal parasites	causes trypanosomiasis, flagellate parasite	present	absent	
	Trypanosoma rangeli	0	terrestrial	animal parasites	causes trypanosomiasis, flagellate parasite	present	absent	

**Excavates --> Heterolobozoa**								
	Naegleria gruberi	0	fresh water	heterorophic species	amoeba, free - living organism	present	absent	
**Amoebozoa --> Mycetozoa**								
	Dictyostelium discoideum	0	terrestrial	heterorophic species	soil - living amoeba	present	absent	
	Dictyostelium fasciculatum	0	terrestrial	heterorophic species	soil - living amoeba	present	absent	
	Dictyostelium purpureum	0	terrestrial	heterorophic species	soil - living amoeba	present	absent	
	Polysphondylium pallidum	0	terrestrial	heterorophic species	amoeba	present	absent	
**Amoebozoa --> Archamoebae**								
	Entamoeba dispar	0	terrestrial	animal parasites	pathogenic amoeba, causes amoebiasis, dysentery	present	absent	
	Entamoeba histolytica	1	terrestrial	animal parasites	pathogenic amoeba, causes amoebiasis, dysentery	present	yes	
	Entamoeba invadens	2	terrestrial	animal parasites	pathogenic amoeba, causes amoebiasis, dysentery	present	yes	
	Entamoeba nuttalli	0	terrestrial	animal parasites	pathogenic amoeba, causes amoebiasis, dysentery	present	absent	
**Amoebozoa --> Discosea**								
	Acanthamoeba castellanii	0	fresh water	animal parasites	amoeba, bacterivores, cause acanthamebiasis	present	absent	
**Straminipila**								
	Blastocystis hominis	0	terrestrial	animal parasites	causess stomach problem in humans	present	absent	
**Straminipila --> Synurophyceae**								
	Poterioochromonas sp.	1	freshwater	autotrophic species	heterokont algae, photosynthetic	absent	yes	0
	Synura sp.	1	freshwater	autotrophic species	heterokont algae, photosynthetic	absent	no	0
**Straminipila --> Bacillariophyta**								
	Chaetoceros affinis	1	marine	autotrophic species	diatom (brown algaes), phytoplankton	absent	no	0
	Leptocylindrus danicus	1	marine	autotrophic species	diatom (brown algaes), phytoplankton	absent	no	0
	Nitzschia sp.	1	marine	autotrophic species	diatom (brown algaes), phytoplankton, produces neurotoxin, grow at cold temperature	absent	yes	0
	Phaeodactylum tricornutum	1	marine	autotrophic species	diatom (brown algaes) phytoplankton	present	yes	
	Thalassiosira oceanica	1	marine	autotrophic species	phytoplankton, diatom (brown algaes), symbiosis with bacteria	present	yes	
**Straminipila --> Oomycetes**								
	Albugo candida	0	terrestrial	plant parasites	obligatory biotrophic plant parasite, cause white blister rust	present	absent	
	Aphanomyces astaci	0	terrestrial	animal parasites	crayfish parasites,	present	absent	
	Aphanomyces invadans	0	terrestrial	animal parasites	fish parasite	present	absent	
	Hyaloperonospora arabidopsidis	0	terrestrial	plant parasites	causes mildew	present	absent	
	Phytophthora capsici	4	terrestrial	plant parasites	mildew	absent	yes	58.277
	Phytophthora cinamoni	4	terrestrial	plant parasites	mildew	absent	yes	330.482
	Phytophthora infestans	2	terrestrial	plant parasites	mildew	present	yes	
	Phytophthora kernoviae	2	terrestrial	plant parasites	mildew	present	yes	
	Phytophthora lateralis	3	terrestrial	plant parasites	mildew	present	yes	

	Phytophthora nicotianae	3	terrestrial	plant parasites	cause root rot, crown rot, leaf infection	present	yes	
	Phytophthora parasitica	3	terrestrial	plant parasites	soilborne pathogen	present	yes	
	Phytophthora ramorum	4	terrestrial	plant parasites	causes the disease sudden oak death (SOD)	present	yes	
	Phytophthora sojae	5	terrestrial	plant parasites	causes damping - off	present	yes	
	Plasmopara halstedii	1	terrestrial	plant parasites	causes sunflowers mildew	present	yes	
	Pythium aphanidermatum	4	terrestrial	plant parasites	mildew	present	yes	
	Pythium arrhenomanes	2	terrestrial	plant parasites	mildew	present	yes	
	Pythium irregulare	2	terrestrial	plant parasites	mildew	present	yes	
	Pythium iwayamai	4	terrestrial	plant parasites	mildew	present	yes	
	Pythium ultimum	4	terrestrial	plant parasites	mildew	present	yes	
	Pythium vexans	4	terrestrial	plant parasites	mildew	present	yes	
	Saprolegnia diclina	0	terrestrial	animal parasites	amphibian and fish parasites, causes oomycosis, secondary pathogen	present	absent	
**Straminipila --> Phaeophyceae**								
	Ectocarpus siliculosus	0	marine	autotrophic species	brown algaes	present	absent	
	Sargassum vulgare	1	marine	autotrophic species	brown algaes	absent	no	0
**Straminipila --> Pelagophyceae**								
	Aureococcus anophagefferens	1	marine	autotrophic species	algaes, causes algal blooms	present	yes	
	Aureoumbra lagunensis	1	marine	heterorophic species	picoplankton	absent	yes	0
**Straminipila --> Eustigmatophyceae**								
	Nannochloropsis gaditana	2	fresh water	autotrophic species	microalgaes, phytoplankton	present	yes	
**Alveolata --> Dinophyceae**								
	Alexandrium tamarense	1	marine	autotrophic species	phytoplankton, algae	absent	no	10.887
	Gambierdiscus excentricus	1	marine	autotrophic species	brown macroalgae, produce toxins, algae	absent	no	0
	Lingulodinium polyedrum	1	marine	autotrophic species	algae, motile dinoflagelllates, production of Yessotoxins (YTXs)	absent	no	3.640
	Perkinsus marimus	0	marine	animal parasites	pathogen of oysters, , algae	present	absent	
	Symbiodinium minutum	2	marine	autotrophic species	endosymbiotic dinoflagellates, alguae	present	yes	
**Alveolata --> Apicomplexa**								
	Babesia bigemina	0	terrestrial	animal parasites	causes babebiosis	present	absent	
	Babesia bovis	0	terrestrial	animal parasites	causes babebiosis	present	absent	
	Cryptosporidium hominis	0	terrestrial	animal parasites	obligate humans parasite, causes cryptosporidiosis	present	absent	
	Eimeria acervulina	1	terrestrial	animal parasites	causes coccidiosis	present	yes	
	Eimeria brunetti	1	terrestrial	animal parasites	causes coccidiosis	present	yes	
	Eimeria maxima	1	terrestrial	animal parasites	causes coccidiosis	present	yes	
	Eimeria mitis	1	terrestrial	animal parasites	causes coccidiosis	present	yes	
	Eimeria necatrix	1	terrestrial	animal parasites	causes coccidiosis	present	yes	
	Eimeria praecox	1	terrestrial	animal parasites	causes coccidiosis	present	yes	
	Eimeria tenella	1	terrestrial	animal parasites	causes coccidiosis	present	yes	
	Gregarina niphandrodes	1	terrestrial	animal parasites	invertebrate parasite	present	yes	
	Hammondia hammondi	1	terrestrial	animal parasites	dogs parasite	present	yes	
	Neospora caninum	1	terrestrial	animal parasites	causes neosporosis	present	yes	
	Plasmodium berghei	1	terrestrial	animal parasites	causes malaria	present	yes	
	Plasmodium chabaudi	1	terrestrial	animal parasites	causes malaria	present	yes	

	Plasmodium cynomolgi	1	terrestrial	animal parasites	causes malaria	present	yes	
	Plasmodium falciparum	1	terrestrial	animal parasites	causes malaria	present	yes	
	Plasmodium fragile	1	terrestrial	animal parasites	causes malaria	present	yes	
	Plasmodium inui	1	terrestrial	animal parasites	causes malaria	present	yes	
	Plasmodium knowlesi	1	terrestrial	animal parasites	causes malaria	present	yes	
	Plasmodium reichenowi	1	terrestrial	animal parasites	causes malaria	present	yes	
	Plasmodium vinckei	1	terrestrial	animal parasites	causes malaria	present	yes	
	Plasmodium vivax	1	terrestrial	animal parasites	causes malaria	present	yes	
	Plasmodium yoelii	1	terrestrial	animal parasites	causes malaria	present	yes	
	Theileria annulata	0	terrestrial	animal parasites	causes theileriosis (east coast fever)	present	absent	
	Theileria orientalis	0	terrestrial	animal parasites	causes theileriosis (east coast fever)	present	absent	
	Theileria parva	0	terrestrial	animal parasites	causes theileriosis (east coast fever)	present	absent	
	Toxoplasma gondii	1	terrestrial	animal parasites	causes toxoplasmosis	present	yes	
**Alveolata --> Chromerida**								
	Vitrella brassicaformis	2	marine	autotrophic species	isolated from the Great Barrier Reef, mixotrophe	present	yes	
**Alveolata --> Ciliophora**								
	Ichthyophthirius multifilis	0	fresh water	animal parasites	fish parasite (causes ichtyophthiriosis)	present	absent	
	Oxytricha trifallax	0	fresh water	heterorophic species	free living, very mobile	present	absent	
	Paramecium tetraurelia	0	fresh water	heterorophic species	phagocytose bacteria	present	absent	
	Stylonichia lemnae	0	fresh water	heterorophic species	free living, carnivorous (eat other protozoans and bacteria)	present	absent	
	Tetrahymena thermophila	0	fresh water	heterorophic species	commensalistic	present	absent	
**Opisthokonts --> Ichthyospora**								
	Capsaspora owczarzaki	0	fresh water	animal parasites	amoeboid, snail parasite and kill the sporocysts of flatworm	present	absent	
**Opisthokonts --> Nucleariids**								
	Fonticula alba	0	terrestrial	heterorophic species	amoeboa - free living	present	absent	
**Opisthokonts --> Choanoflagellata**								
	Monosiga brevicollis	0	marine	heterorophic species	free living, nanoplankton	present	absent	
	Salpingoeca rosetta	1	fresh water	heterorophic species	free living	present	yes	
**Opisthokonts --> Dikarya --> Basidiomycota --> Pucciniomycotina**								
	Agaricostilbum hyphaenes	0	terrestrial	heterorophic species	occurs commonly on palm litter, saprophytic fungi	present	absent	
	Chionosphaera apobasidialis	0	terrestrial	plant parasites	parasite of lichens	present	absent	
	Cronartium quercuum	0	terrestrial	plant parasites	causes plant diseases of pinus	present	absent	
	Erythrobasidium hasegawianum	0	terrestrial	heterorophic species	saprophytic fungi	present	absent	
	Heterogastridium pycnidioideum	0	terrestrial	animal parasites	fungi parasites	present	absent	
	Hyalopycnis blepharistoma	0	terrestrial	heterorophic species	saprophytic fungi	present	absent	
	Leucosporidiella creatinivora	0	terrestrial	heterorophic species	saprophytic fungi, isolated from permafrost soil	present	absent	
	Melampsora laricis	0	terrestrial	plant parasites	most devastating and widespread pathogen of poplars	present	absent	
	Meredithblackwellia eburnea	0	terrestrial	heterorophic species	saprophytic fungi	present	absent	

	Mixia osmundae	0	terrestrial	plant parasites	intracellular parasite of ferns, obligatory plant pathogens	present	absent	
	Naiadella fluitans	0	fresh water	animal parasites	mycoparasitic	present	absent	
	Naohidea sebacea	0	terrestrial	animal parasites	mycoparasitic	present	absent	
	Puccinia graminis	0	terrestrial	plant parasites	causes stem rusts, obligatory plant pathogens	present	absent	
	Puccinia triticina	0	terrestrial	plant parasites	causes wheat leaf rust, obligatory plant pathogens	present	absent	
	Rhodosporidium toruloides	0	terrestrial	animal parasites	isolated from wood pulp from conifers, link to bovine mastitis	present	absent	
	Rhodotorula graminis	0	terrestrial	animal parasites	causes infections in animals	present	absent	
	Rhodotorula minuta	0	terrestrial	animal parasites	human parasite	present	absent	
	Septobasidium sp.	0	terrestrial	animal parasites	entomopathogens	present	absent	
	Sporobolomyces pararoseus	0	terrestrial	heterorophic species	saprophytic fungi	present	absent	
**Dikarya --> Basidiomycota --> Ustilaginomycotina**								
	Acaromyces ingoldii	0	terrestrial	heterorophic species	causes mites mortality, saprophytic fungi	present	absent	
	Ceraceosorus guamensis	0	terrestrial	heterorophic species	saprophytic fungi	present	absent	
	Exobasidium vaccinii	0	terrestrial	plant parasites	plant pathogen that grow on Ericaceae	present	absent	
	Jaminaea sp.	0	terrestrial	heterorophic species	saprophytic fungi	present	absent	
	Malassezia globosa	0	terrestrial	animal parasites	opportunistic pathogens of humans, causes skin disorders	present	absent	
	Malassezia sympodialis	0	terrestrial	animal parasites	opportunistic pathogens of humans, causes skin disorders	present	absent	
	Meira mitonrushii	0	terrestrial	heterorophic species	saprophytic fungi, endophyte	present	absent	
	Melanotaenium endogenum	0	terrestrial	plant parasites	Bedstraw smut, pathogen of Galium mollugo	present	absent	
	Pseudozyma antarctica	0	terrestrial	heterorophic species	saprophytic fungi	present	absent	
	Pseudozyma hubeiensis	0	terrestrial	plant parasites	epiphytic fungus	present	absent	
	Sporisorium reilanum	0	terrestrial	plant parasites	Head Smut pathogen of Sorghum	present	absent	
	Testicularia cyperi	0	terrestrial	plant parasites	colonizes Rhynchospora sp, plant pathogens	present	absent	
	Tilletiaria anomala	0	terrestrial	heterorophic species	saprophytic fungi - decayed wood	present	absent	
	Tilletiopsis washingtonensis	0	terrestrial	heterorophic species	saprophytic fungi	present	absent	
	Ustilago maydis	0	terrestrial	plant parasites	plant pathogen, causes corn smut	present	absent	
**Dikarya --> Basidiomycota --> Agaricomycotina --> Agaricales**								
	Agaricus bisporus	1	terrestrial	heterorophic species	portobello mushroom, saprophytic fungi	present	yes	
	Amanita muscaria	1	terrestrial	heterorophic species	fly agaric or fly amanita, mycorrhizal	present	yes	
	Armillaria mellea	1	terrestrial	plant parasites	honney fungus, plant pathogen	absent	yes	30
	Coprinopsis cinerea	1	terrestrial	heterorophic species	saprophytic fungi, gray shap	present	yes	
	Cortinarius glaucopus	0	terrestrial	plant parasites	saprophytic fungi, blue-foot webcap, ectomycorrhizal	present	absent	
	Fistulina hepatica	1	terrestrial	plant parasites	beefsteack fungus, parasitic	present	yes	

	Galerina marginata	1	terrestrial	plant parasites	saprophytic fungi - ectomyccorrhizal - produces amatoxins	present	yes	
	Gymnopus luxurians	1	terrestrial	heterorophic species	saprophytic fungi	present	yes	
	Hebeloma cylindrosporum	1	terrestrial	plant parasites	saprophytic fungi - ectomycchorhizal	present	yes	
	Hypholoma sublateritium	1	terrestrial	heterorophic species	saprophytic fungi, brick cap	present	yes	
	Hypsizygus marmoreus	1	terrestrial	heterorophic species	saprophytic fungi	present	yes	
	Laccaria amethystina	1	terrestrial	plant parasites	amethyst deceiver, mycorrhizal	present	yes	
	Laccaria bicolor	1	terrestrial	plant parasites	ectomycorrhizal	present	yes	
	Leucoagaricus gongylophorus	1	terrestrial	heterorophic species	saprophytic fungi - associates with ants	present	yes	
	Panellus stipticus	0	terrestrial	heterorophic species	saprophytic fungi, bitter oyster	present	absent	
	Pleurotus ostreatus	1	terrestrial	heterorophic species	saprophytic fungi - white -rot wood decay fungus	present	yes	
	Rhodocollybia butyracea	0	terrestrial	heterorophic species	saprophytic fungi, butterfly collybia	present	absent	
	Schizophyllum commune	1	terrestrial	heterorophic species	saprophytic fungi	present	yes	
	Termitomyces sp. J132	1	terrestrial	heterorophic species	saprophytic fungi - food source for termites	present	no	
**Dikarya --> Basidiomycota --> Agaricomycotina --> Atheliales**								
	Piloderma croceum	1	terrestrial	plant parasites	ectomyccorrhizal	present	yes	
**Dikarya --> Basidiomycota --> Agaricomycotina --> Auriculariales**								
	Auricularia subglabra	1	terrestrial	heterorophic species	saprophytic fungi, produce a white rot on dead wood	present	yes	
**Dikarya --> Basidiomycota --> Agaricomycotina --> Polyporales**								
	Bjerkandera adusta	1	terrestrial	plant parasites	saprophytic fungi, smoky polypore, plant pathogen, cause white rot in live trees	present	yes	
	Ceriporiopsis subvermispora	1	terrestrial	heterorophic species	saprophytic fungi, crust fungi that cause a white rot	present	yes	
	Daedalea quercina	1	terrestrial	heterorophic species	saprophytic fungi, oak mazegill, causes brown rot	present	yes	
	Dichomitus squalens	1	terrestrial	heterorophic species	saprophytic fungi	present	yes	
	Exidia glandulosa	1	terrestrial	heterorophic species	black jelly roll, saprophytic fungi	present	yes	
	Fibroporia radiculosa	0	terrestrial	heterorophic species	saprophytic fungi - brown rot fungus	present	absent	
	Fibulorhizoctonia sp.	0	terrestrial	heterorophic species	cuckoo fungus, saprophytic fungis, use by termite	present	absent	
	Fomitopsis pinicola	1	terrestrial	heterorophic species	stem decay fungus, red belt conk, saprophytic fungi	present	yes	
	Ganoderma lucidum	1	terrestrial	heterorophic species	saprophytic fungi, grow on both coniferous and hardwood species	present	yes	
	Gelatoporia subvermispora	1	terrestrial	heterorophic species	saprophytic fungi	present	yes	
	Laetiporus sulphureus	1	terrestrial	heterorophic species	sulphur polyphore, saprophytic fungi	present	yes	
	Lentinus tigrinus	1	terrestrial	heterorophic species	saprophytic fungi	present	yes	
	Phanerochaete carnosa	1	terrestrial	plant parasites	plant pathogen that infects plane trees	present	yes	
	Phlebiopsis gigantea	1	terrestrial	heterorophic species	saprophytic fungi, causes white rot on conifer	present	yes	

	Plicaturopsis crispa	1	terrestrial	heterorophic species	saprophytic fungi	present	yes	
	Polyporus arcularius	1	terrestrial	heterorophic species	causes white rot, saprophytic fungi	present	yes	
	Postia placenta	1	terrestrial	heterorophic species	saprophytic fungi, causes brown rot	present	yes	
	Pycnoporus cinnabarinus	1	terrestrial	plant parasites	white rot saprobic fungus, plant pathogen infecting plane and mango trees	present	yes	
	Trametes cinnabarina	1	terrestrial	heterorophic species	saprophytic fungi	present	yes	
	Trametes versicolor	1	terrestrial	heterorophic species	turkey tail, saprophytic fungi	present	yes	
	Wolfiporia cocos	2	terrestrial	heterorophic species	hoelen, saprophytic fungi	present	yes	
**Dikarya --> Basidiomycota --> Agaricomycotina --> Boletales**								
	Boletus edulis	0	terrestrial	plant parasites	penny bun, formes ectomycorrhizal associations with living trees	present	absent	
	Coniophora puteana	1	terrestrial	heterorophic species	saprophytic fungi - causes wet root in wood	present	yes	
	Gyrodon lividus	0	terrestrial	plant parasites	the alder bolete, mycorrhizal association with alder	present	absent	
	Hydnomerulius pinastri	1	terrestrial	heterorophic species	saprophytic fungi, causes a brown - rot, called the spiny dry rot fungus	present	yes	
	Paxillus involutus	1	terrestrial	plant parasites	saprophytic fungicommonly known as the brown roll-rim, ectomycorrhizal	present	yes	
	Paxillus rubicundulus	1	terrestrial	plant parasites	ectomycorrhizal	present	yes	
	Pisolithus tinctorius	1	terrestrial	plant parasites	mycchorrhizal, also know as the dyemaker's puffball	present	yes	
	Rhizopogon salebrosus	0	terrestrial	plant parasites	false truffles, ectomycorrhizal	present	absent	
	Serpula lacrymans	1	terrestrial	heterorophic species	saprophytic fungi - causes dry rot - most damaging destroyer of indoor wood	present	yes	
	Suillus granulatus	0	terrestrial	plant parasites	ectomycorrhizal, weeping or granulated bolete	present	absent	
	Suillus hirtellus	0	terrestrial	plant parasites	ectomycorrhizal	present	absent	
	Suillus luteus	0	terrestrial	plant parasites	saprophytic fungi, slippery jack or sticky bun, ectomycorrhizal	present	absent	
	Suillus pictus - spraguei	0	terrestrial	plant parasites	painted slipperycap, mycorrhizal	present	absent	
**Dikarya --> Basidiomycota --> Agaricomycotina --> Corticiales**								
	Cylindrobasidium torrendii	1	terrestrial	heterorophic species	saprophytic fungi	present	yes	
	Punctularia strigosozonata	1	terrestrial	heterorophic species	saprophytic fungi, causes a white rot	present	yes	
	Schizopora paradoxa	1	terrestrial	heterorophic species	saprophytic fungi - decayed frondose wood	present	yes	
**Dikarya --> Basidiomycota --> Agaricomycotina --> Hymenochaetales**								
	Fomitiporia mediterranea	1	terrestrial	plant parasites	cause esca of grapewine	present	yes	
**Dikarya --> Basidiomycota --> Agaricomycotina --> Gloephyllales**								
	Gloeophyllum trabeum	1	terrestrial	heterorophic species	brown rot fungus, saprophytic fungi	present	yes	
	Neolentinus lepideus	1	terrestrial	heterorophic species	scaly lentinus, saprophytic fungi	present	yes	
**Dikarya --> Basidiomycota --> Agaricomycotina --> Russulales**								

	Heterobasidion irregulare	1	terrestrial	plant parasites	tree root rotting pathogenic fungus	present	yes	
	Peniophora sp.	1	terrestrial	plant parasites	plant pathogens	present	yes	
	Stereum hirsutum	1	terrestrial	plant parasites	false turkey tail, plant pathogen infecting peach trees	present	yes	
**Dikarya --> Basidiomycota --> Agaricomycotina --> Jaapiales**								
	Jaapia argillacea	2	terrestrial	heterorophic species	saprophytic fungi, produces a type of brown rot	present	yes	
**Dikarya --> Basidiomycota --> Agaricomycotina --> Gomphales**								
	Ramaria rubella	1	terrestrial	plant parasites	mycorrhizal, red coral	present	yes	
**Dikarya --> Basidiomycota --> Agaricomycotina --> Geastrales**								
	Sphaerobolus stellatus	1	terrestrial	heterorophic species	saprophytic fungi, shotgun or cannonball fungus	present	yes	
**Dikarya --> Basidiomycota --> Agaricomycotina --> Tremellomycetes**								
	Cryptococcus gattii	0	terrestrial	animal parasites	cause cryptococcosis	present	absent	
	Cryptococcus neoformans	0	terrestrial	animal parasites	cause cryptococcosis	present	absent	
	Dioszegia cryoxerica	0	terrestrial	heterorophic species	psychrophilic yeasts from polar desert soils	present	absent	
	Fellomyces penicillatus	0	terrestrial	heterorophic species	saprophytic fungi	present	absent	
	Fibulobasidium inconspicuum	0	terrestrial	heterorophic species	saprophytic fungi	present	absent	
	Sistotremastrum suecicum	0	terrestrial	heterorophic species	saprophytic fungi, cause a brown rot of various tree species	present	absent	
	Tremella enphala	0	terrestrial	animal parasites	parasitic on another species of fungus	present	absent	
	Tremella mesenterica	0	terrestrial	animal parasites	yellow brain, parasite of wood decay fungi	present	absent	
	Trichosporon oleaginosus	0	terrestrial	heterorophic species	saprophytic fungi	present	absent	
**Dikarya --> Basidiomycota --> Agaricomycotina --> Cantharellales**								
	Botryobasidium botryosum	0	terrestrial	heterorophic species	saprophytic fungis	present	absent	
	Cantharelus anzutake	0	terrestrial	plant parasites	golden chanterelle, ectomycorrhizal	present	absent	
	Clavulina sp.	0	terrestrial	plant parasites	saprophytic fungis - ectomycorrhizal	present	absent	
	Hydnum rufescens	0	terrestrial	plant parasites	symbiotic fungi identified in orchids	present	absent	
	Rhizoctonia solani	1	terrestrial	plant parasites	plant pathogenus fungi, causes collar and root rot	present	yes	
	Tulasnella calospora	0	terrestrial	plant parasites	mychorhizien	present	absent	
**Dikarya --> Basidiomycota --> Agaricomycotina --> Wallemiomycetes**								
	Wallemia ichthyophaga	0	terrestrial	heterorophic species	halophilic - saprophytic fungi	present	absent	
	Wallemia sebi	0	terrestrial	heterorophic species	xerophilic - saprophytic fungi	present	absent	
**Dikarya --> Basidiomycota --> Agaricomycotina --> Dacrymycetales**								
	Calocera cornea	0	terrestrial	heterorophic species	saprophytic fungi - grows on decaying wood	present	absent	
	Calocera viscosa	0	terrestrial	heterorophic species	saprophytic fungi - yellow stagshorn	present	absent	
	Dacryopinax primogenitus	0	terrestrial	heterorophic species	saprophytic fungi - causes extensive decay in dead wood	present	absent	
**Dikarya --> Ascomycota --> Pezizomycotina --> Pezizomycetes**								
	Ascobolus immersus	0	terrestrial	heterorophic species	coprophilous	present	absent	
	Ascodesmis nigricans	0	terrestrial	heterorophic species	coprophilous	present	absent	
	Caloscypha fulgens	0	terrestrial	plant parasites	saprophytic fungi - infect dormant seeds of Picea sitchensis - spring orange peel fungus	present	absent	

	Choiromyces venosus	0	terrestrial	plant parasites	ectomyccorrhizal - pig truffle	present	absent	
	Gyromitra esculenta	0	terrestrial	heterorophic species	saprophytic fungi	present	absent	
	Kalaharituber pfeilii	0	terrestrial	plant parasites	saprophytic fungi - ectomyccorrhizal	present	absent	
	Morchella importuna	0	terrestrial	heterorophic species	saprophytic fungi	present	absent	
	Plectania melastoma	0	terrestrial	heterorophic species	saprophytic fungi (neither mycorrhizal nor edible)	present	absent	
	Pyronema confluens	0	terrestrial	heterorophic species	saprophytic fungi	present	absent	
	Sarcoscypha coccinea	0	terrestrial	heterorophic species	saprophytic fungi - scarlet elf cup	present	absent	
	Terfezia boudieri	0	terrestrial	plant parasites	saprophytic fungi - ectomycorrhizal - desert truffe	present	absent	
	Tuber melanosporum	0	terrestrial	plant parasites	saprophytic fungi - ectomycorrhizal- black truffle	present	absent	
	Wilcoxina mikolae	0	terrestrial	plant parasites	ectomycorrhizal	present	absent	
**Dikarya --> Ascomycota --> Pezizomycotina --> Orbiliomycetes**								
	Arthrobotrys oligospora	0	terrestrial	heterorophic species	carnivorus fungus (nematode-trapping)	present	absent	
	Dactylellina haptotyla	0	terrestrial	heterorophic species	carnivorus fungus (nematode-trapping)	present	absent	
	Monacrosporium haptotylum	0	terrestrial	heterorophic species	carnivorus fungus (nematode-trapping)	present	absent	
**Dikarya --> Ascomycota --> Pezizomycotina --> Eurotiomycetes**								
	Ajellomyces capsulatus	1	terrestrial	animal parasites	cause histoplasmosis	present	yes	
	Ajellomyces dermatitidis	1	terrestrial	animal parasites	cause histoplasmosis	present	yes	
	Arthroderma otae	0	terrestrial	animal parasites	anthropophilic saprotrop, colonizes the dead skin, fungal infection of nail	present	absent	
	Aspergillus sp.	0	terrestrial	heterorophic species	saprophytic fungi	present	absent	
	Aspergillus sydowii	0	terrestrial	animal parasites	cause of aspergillosis in sea fan corals	present	absent	
	Blastomyces gilchristii	1	terrestrial	animal parasites	human parasites, causes blastomycosis	present	yes	
	Emmonsia crescens	1	terrestrial	animal parasites	causes adiaspiromycosis	present	yes	
	Emmonsia parva	1	terrestrial	animal parasites	causes adiaspiromycosis	present	yes	
	Eurotium rubrum	0	terrestrial	heterorophic species	saprophytic fungi - live in Dead Sea water	present	absent	
	Gymnascella aurantiaca	0	terrestrial	heterorophic species	saprophytic fungi	present	absent	
	Gymnascella citrina	0	terrestrial	heterorophic species	saprophytic fungi	present	absent	
	Histoplasma capsulatum	1	terrestrial	animal parasites	causes histoplasmosis - opportunist human pathogen	present	yes	
	Monascus ruber	0	terrestrial	heterorophic species	corn silage mold	present	absent	
	Paracoccidioides brasiliensis	1	terrestrial	animal parasites	causes paracoccidioidomycosis	present	yes	
	Paracoccidioides lutzii	1	terrestrial	animal parasites	causes paracoccidioidomycosis	present	yes	
	Penicilium oxalicum	0	terrestrial	heterorophic species	saprophytic fungi	present	absent	
	Talaromyces marneffei	0	terrestrial	animal parasites	opportunistic human pathogen	present	absent	
	Thermoascus aurantiacus	0	terrestrial	heterorophic species	thermophilic fungus, saprophytic fungi	present	absent	

	Trichophyton rubruma	0	terrestrial	animal parasites	anthropophilic saprotrop, colonizes the dead skin, fungal infection of nail	present	absent	
**Dikarya --> Ascomycota --> Pezizomycotina --> Dothideomycetes**								
	Acidomyces richmondensis	0	terrestrial	heterorophic species	acidophilic filamentous fungi	present	absent	
	Ascophyta	0	terrestrial	plant parasites	pathogene of plants (particularly cereal crops)	present	absent	
	Aureobasidium pullulans	0	terrestrial	plant parasites	epiphyte, endophyte, pathogen opportunist of humans	present	absent	
	Bipolaris oryzae	0	terrestrial	plant parasites	causes brown spot disease in rice	present	absent	
	Dothistroma septosporum	0	terrestrial	plant parasites	causes red band needle blight	present	absent	
	Leptosphaeria maculans	0	terrestrial	plant parasites	causal agent of blackleg disease	present	absent	
	Macrophomina phaseolina	0	terrestrial	plant parasites	causes damping off, seedling blight and collar rot	present	absent	
	Neofusicoccum parvum	0	terrestrial	plant parasites	causing serious damage to Eucalyptus	present	absent	
	Parastagonospora nodorum	0	terrestrial	plant parasites	major fungal pathogen of wheat	absent	absent	15.973
	Pseudocercospora fijiensis	0	terrestrial	plant parasites	plant pathogens	present	absent	
	Verruconis gallopava	0	terrestrial	animal parasites	Human parasitic phaeohyphomycosis	present	absent	
	Zymoseptoria brevis	1	terrestrial	plant parasites	causes septoria leaf blotch	present	no	
**Dikarya --> Ascomycota --> Pezizomycotina --> Lecanoromycetes**								
	Xanthoria parietina	0	terrestrial	heterorophic species	lichens	present	absent	
**Dikarya --> Ascomycota --> Pezizomycotina --> Leotiomycetes**								
	Acephala macrosclerotiorum	0	terrestrial	heterorophic species	saprophytic fungi - forms ectomycorrhizas on Pinus sylvestris	present	absent	
	Amorphotheca resinae	0	terrestrial	heterorophic species	saprophytic fungi - can live in tanks of diesel	present	absent	
	Blumeria graminis	0	terrestrial	plant parasites	causes barley powdery mildew on grasses including cereals	present	absent	
	Botrytis cinerea	0	terrestrial	plant parasites	necrotrophic fungus, botrytis bunch rot	present	absent	
	Bulgaria inquinans	0	terrestrial	heterorophic species	saprophytic fungi - black bulgar	present	absent	
	Cadophora malorum	0	terrestrial	plant parasites	plant pathogen, causes side rot in apple and pear	present	absent	
	Chalara longipes	0	terrestrial	heterorophic species	saprophytic fungi	present	absent	
	Coccomyces strobi	0	terrestrial	plant parasites	plant pathogen - causes Cherry leaf spot	present	absent	
	Erysiphe necator	0	terrestrial	plant parasites	causes powdery mildew of grape	present	absent	
	Leptodontium sp.	0	terrestrial	heterorophic species	saprophytic fungi - responsible for Sooty Blotch disease	present	absent	
	Loramyces juncicola	0	terrestrial	heterorophic species	saprophytic fungi	present	absent	
	Loramyces macrosporus	0	terrestrial	heterorophic species	saprophytic fungi	present	absent	
	Meliniomyces bicolor	0	terrestrial	plant parasites	saprophytic fungi - ectomyccorrhizal - root-associated fungi	present	absent	
	Meliniomyces variabilis	0	terrestrial	plant parasites	saprophytic fungi - ectomyccorrhizal - root-associated fungi	present	absent	
	Oidiodendron maius	0	terrestrial	heterorophic species	mycorrhizal fungus	present	absent	

	Phialocephala scopiformis	0	terrestrial	plant parasites	endophyte	present	absent	
	Rhizoscyphus ericae	0	terrestrial	plant parasites	mycorrhizal fungus	present	absent	
	Rutstroemia firma	0	terrestrial	heterorophic species	saprophytic fungi - grows on wood	present	absent	
	Sclerotinia borealis	0	terrestrial	plant parasites	cause a plant pathogen infecting barley	present	absent	
	Sclerotinia sclerotiorum	0	terrestrial	plant parasites	cause a disease called white mold	present	absent	
	Sporothrix insectorum	1	terrestrial	heterorophic species	decomposing plant material and pathogen oppportunist (causative agent of sporotrichosis)	present	yes	
	Thelebolus microsporus	0	terrestrial	heterorophic species	saprobic fungus	present	absent	
**Dikarya --> Ascomycota --> Pezizomycotina --> Sordariomycetes**								
	Acremonium alcalophilum	0	terrestrial	heterorophic species	saprophytic fungi	present	absent	
	Anthostoma avocetta	0	terrestrial	heterorophic species	saprophytic fungi	present	absent	
	Apiospora montagnei	0	terrestrial	plant parasites	causes kernel blight on barley	present	absent	
	Ceratocystis sp.	0	terrestrial	plant parasites	plant pathogens, causing diseases such as oak wilt and pineapple black rot	absent	absent	3.432
	Clonostachys rosea	0	terrestrial	animal parasites	endophyte, saprophytic fungi, parasite of other fungi and of nematodes	present	absent	
	Colletotrichum caudatum	0	terrestrial	plant parasites	phytopathogens	present	absent	
	Colletotrichum zoysiae	0	terrestrial	plant parasites	most common and destructive of plant pathogenic fungi	present	absent	
	Coniochaeta sp.	0	terrestrial	plant parasites	saprotrophs, endophytes and pathogens of trees	present	absent	
	Corollospora maritima	0	marine	heterorophic species	saprophytic fungi	present	absent	
	Cryphonectria parasitica	0	terrestrial	plant parasites	causes chestnut blight	present	absent	
	Daldinia eschscholzii	0	terrestrial	heterorophic species	saprotrophic	present	absent	
	Drechmeria coniospora	0	terrestrial	animal parasites	entomopathons	absent	absent	0
	Fusarium sp.	0	terrestrial	heterorophic species	saprophytic fungis - produces mycotoxins in cereal crops	present	absent	
	Glomerella graminicola	0	terrestrial	plant parasites	causes anthracnose in many cereal	present	absent	
	Hypoxylon sp.	0	terrestrial	heterorophic species	saprophytic fungi, found on dead wood	present	absent	
	Ilyonectria sp.	0	terrestrial	plant parasites	symptoms of root rot on herbaceous plants	present	absent	
	Lindra thalassiae	0	marine	plant parasites	parasites of various sea grasses	present	absent	
	Lollipopaia minuta	0	terrestrial	heterorophic species	saprophytic fungi	present	absent	
	Magnaporthe orysae	0	terrestrial	plant parasites	also known as rice blast fungus, rice rotten neck	present	absent	
	Melanconium juglandinum	0	terrestrial	plant parasites	plant pathogen	present	absent	
	Melanospora tiffanyae	0	terrestrial	animal parasites	mycoparasitic fungus	present	absent	
	Microascus trigonosporus	0	terrestrial	animal parasites	saprotrophic, opportunistic human pathogen	present	absent	

	Microdochium bolleyi	0	terrestrial	plant parasites	plant pathogen - causes root rot in flax and wheat	present	absent	
	Myceliophthora thermophila	0	terrestrial	heterorophic species	saprophytic fungi	present	absent	
	Myrothecium inundatum	0	terrestrial	heterorophic species	saprotrophic, endophyte	present	absent	
	Neurospora crassa	0	terrestrial	heterorophic species	model organism, can survive on minimal media	present	absent	
	Niesslia exilis	0	terrestrial	heterorophic species	saprotrophic	present	absent	
	Periconia macrospina	0	terrestrial	heterorophic species	plant symbiotic species	present	absent	
	Plectosphaerella cucumerina	0	terrestrial	plant parasites	pathogen of several plant causing fruit, root and collar rot	absent	absent	0
	Podospora curvicolla	0	terrestrial	heterorophic species	coprophilous	present	absent	
	Pseudogymnoascus	0	terrestrial	heterorophic species	saprophytic fungi	present	absent	
	Sodiomyces alkalinus	0	terrestrial	heterorophic species	saprophytic fungi	present	absent	
	Sordaria macrospora	0	terrestrial	heterorophic species	saprophytic fungi - coprophytic species	absent	absent	27
	Thielavia antarctica	0	terrestrial	heterorophic species	coprophilous	present	absent	
	Thielavia terrestris	0	terrestrial	animal parasites	can cause infections in humans	present	absent	
	Thozetella sp.	0	terrestrial	plant parasites	root endophyte of Populus	present	absent	
	Torpedospora radiata	0	terrestrial	heterorophic species	saprobic and cellulocytic fungi	present	absent	
	Trichoderma reesei	0	terrestrial	animal parasites	saprotrophic, pathogens of fungi	present	absent	
	Valetoniellopsis laxa	0	terrestrial	heterorophic species	saprotrophic	present	absent	
**Dikarya --> Ascomycota --> Pezizomycotina --> Xylonomycetes**								
	Symbiotaphrina buchneri	0	terrestrial	animal parasites	endosymbiote of beetles	present	absent	
	Trinosporium guianense	0	terrestrial	heterorophic species	live in association with a tropical tree	present	absent	
	Xylona heveae	0	terrestrial	plant parasites	endophyte of rubber trees	present	absent	
**Dikarya --> Ascomycota --> Pezizomycotina --> Saccharomycotina**								
	Candida albicans	0	terrestrial	animal parasites	cause candidiasis or thrush in humans	present	absent	
	Candida tenuis	0	terrestrial	heterorophic species	saprophytic fungi - ferment xylose	present	absent	
	Eremothecium gossypii	0	terrestrial	plant parasites	cause stigmatomycosis	present	absent	
	Lachancea quebecensis	0	terrestrial	heterorophic species	associated with maple, oak and birch	present	absent	
	Lachancea thermotolerans	0	terrestrial	heterorophic species	usually associated with fruits and Drosophila	present	absent	
	Millerozyma farinosa	0	terrestrial	animal parasites	rare cases of pathogenic on humans	absent	absent	0
	Scheffersomyces stipitis	0	terrestrial	heterorophic species	saprophytic fungi - ferment xylose to ethanol	present	absent	
	Spathaspora passalidatum	0	terrestrial	heterorophic species	study for this ability to assimilate xylose	present	absent	
	Yarrowia lipolytica	0	terrestrial	heterorophic species	saprophytic fungi - can use hydrocarbons	present	absent	
**Dikarya --> Ascomycota --> Pezizomycotina --> Taphrinomycotina**								
	Protomyces lactucaedelis	0	terrestrial	plant parasites	forms galls on different plants	present	absent	
	Saitoella complicata	2	terrestrial	heterorophic species	saprophytic fungi, feeds on decaying matter	present	no	
	Schizosaccharomyces pombe	0	terrestrial	heterorophic species	model organism	present	absent	
**Glomeromycota**								

	Rhizophagus irregularis	1	terrestrial	plant parasites	mycorrhizal fungus	present	yes	
**Mortierellomycotina**								
	Mortierella sp.	0	terrestrial	heterorophic species	saprotrophs in soil	present	absent	
**Mucoromycotina**								
	Absidia glauca	1	terrestrial	animal parasites	causes zygomycosis and mucormycosis	absent	yes	3
	Absidia repens	1	terrestrial	animal parasites	causes zygomycosis and mucormycosis	present	yes	
	Backusella circina	0	terrestrial	heterorophic species	thermophilic fungus	present	absent	
	Blakeslea trispora	1	terrestrial	plant parasites	pathogen autotrophic species, source of beta carotene	present	yes	
	Hesseltinella vesiculosa	0	terrestrial	plant parasites	saprophytic fungis or facultative plant parasites or facultative pathogens of humans (mucoromycotina)	present	absent	
	Lichtheimia corymbifera	1	terrestrial	animal parasites	saprotrophic mold and opportunistic animals pathogen	present	yes	
	Lichtheimia ramosa	1	terrestrial	animal parasites	saprotrophic mold and opportunistic animals pathogen	absent	yes	0
	Mucor ambiguus	1	terrestrial	plant parasites	plant pathogen	absent	yes	0
	Mucor circinelloides	1	terrestrial	plant parasites	plant pathogen	present	yes	
	Parasitella parasitica	1	terrestrial	animal parasites	mycoparasite	present	yes	
	Phycomyces blakesleeanus	1	terrestrial	heterorophic species	saprophytic fungi	present	yes	
	Rhizopus delemar	1	terrestrial	plant parasites	infecting carrots, pineapples, …	present	yes	
	Rhizopus microsporus	1	terrestrial	plant parasites	infecting maize, sunflower, ,,,	present	yes	
	Syncephalastrum racemostrum	1	terrestrial	heterorophic species	nopathogenic organism, common species in soil	present	yes	
	Thamnidium elegans	0	terrestrial	animal parasites	saprophytic fungi mucuromycete	present	absent	
	Umbelopsis ramanniana	1	terrestrial	plant parasites	cosmopolitan saprotrop and endophyte of healthy and declining conifers	present	yes	
**Entomophthoromycotina**								
	Basidiobolus meristosporus	1	terrestrial	animal parasites	human pathogens - commensal in frogs	present	no	
**Blastocladiomycota**								
	Allomyces magrogynus	0	terrestrial	heterorophic species	-	present	absent	
	Catenaria anguillulae	0	terrestrial	animal parasites	facultative parasite of nematode parasites	present	absent	
**Chytridiomycota**								
	Batrachochytrium dendrobatidis	0	terrestrial	animal parasites	amphibian chytrid fungus	present	absent	
	Gonapodya prolifera	1	fresh-water	heterorophic species	carnivorus	present	no	
	Spizellomyces punctatus	0	terrestrial	animal parasites	parasites of organisms that attack nematodes	present	absent	
**Neocallimastigomycota**								
	Piromyces finnis	1	terrestrial	heterorophic species	obligate anaerobes	present	yes	
**Microsporidia**								
	Anncaliia algerae	0	fresh-water	animal parasites	infects mosquitoes	present	absent	
	Anncaliia algerae	0	terrestrial	animal parasites	infects mosquitoes	present	absent	
	Antonospora locustae	0	terrestrial	animal parasites	obligate intracellular parasites	present	absent	
	Encephalitozoon intestinalis	0	terrestrial	animal parasites	cause microsporidiosis	present	absent	
	Encephalitozoon romaleae	0	terrestrial	animal parasites	obligate intracellular parasites	present	absent	

	Nematocida parisii	0	terrestrial	animal parasites	nematode-killer from Paris	present	absent	
	Nosema ceranae	0	terrestrial	animal parasites	parasite of Apis cerana, the Asiatic honey bee	present	absent	
	Ordospora colligata	0	terrestrial	animal parasites	obligatory intracellular parasite	absent	absent	0
**Cryptomycota**								
	Rozella allomycis	0	terrestrial	animal parasites	obligate endoparasite of the water mold	present	absent	
**Opisthokonts --> Metazoa --> Diploblasta --> Placozoa**								
	Trichoplax adhaerens	2	terrestrial	heterorophic species	very flat organisms	present	yes	
**Diploblasta --> Porifera**								
	Amphimedon queenslandica	0	marine	heterorophic species	sponge	present	absent	
**Diploblasta --> Ctenophora**								
	Mnemiopsis leidyi	1	marine	heterorophic species	warty comb jelly or sea walnut	present	yes	
**Diploblasta --> Cnidaria**								
	Hydra magnipapillata	1	fresh water	heterorophic species	hydra	present	yes	
	Nematostella vectensis	1	marine	heterorophic species	Starlet sea anemone	present	yes	
**Opisthokonts --> Metazoa --> Bilateria --> Deuterostomia --> Hemichordata**								
	Saccaglossus kowalevskii	1	marine	heterorophic species	Acorn worm	present	yes	
**Bilateria --> Deuterostomia --> Echinodermata**								
	Paracentrotus lividus	1	marine	heterorophic species	Sea urchin	absent	yes	141217
	Patiria miniata	1	marine	heterorophic species	Blue bat star	present	yes	
	Strongylocentrotus purpuratus	2	marine	heterorophic species	Sea urchin	present	yes	
**Bilateria --> Deuterostomia --> Chordata Urochordates**								
	Ciona intestinalis	2	marine	heterorophic species	Vase tunicate	present	yes	
**Cephalochordates**								
	Branchiostoma floridae	1	marine	heterorophic species	Florida lancelet	present	yes	
**Cyclostomes**								
	Myxine glutinosa	1	marine	heterorophic species	Atlantic hagfish	present	yes	
	Petromyzon marinus	1	marine	heterorophic species	Sea lamprey	present	yes	
**Cartilaginous fish**								
	Callorhinchus milli	2	marine	heterorophic species	Australian ghostshark	present	yes	
**Bony fish**								
	Cyprinus carpio	2	marine	heterorophic species	Common carp	present	yes	
	Dicentrarchus labrax	1	marine	heterorophic species	European bass	present	yes	
	Fundulus heteroclitus	2	marine	heterorophic species	Mummichog	present	yes	
	Haplochromis chilotes	1	fresh-water	heterorophic species	cichlid (fish)	absent	no	35763
	Osmerus mordax	2	marine	heterorophic species	Rainbow smelt	present	yes	
	Salmo salar	2	marine	heterorophic species	Atlantic salmon	present	yes	
	Takifugu rubripes	2	marine	heterorophic species	Japanese puffer	present	yes	
	Xiphophorus maculatus	2	marine	heterorophic species	Southern platyfish	present	yes	
**Amphibians**								
	Andrias davidianus	1	terrestrial	heterorophic species	Chinese giant salamander	absent	yes	1504
	Nanorana parkeri	2	terrestrial	heterorophic species	High Himalaya frog	present	yes	
	Rana catesbeiana	2	terrestrial	heterorophic species	American bullfrog	absent	yes	29138

	Xenopus laevis	2	terrestrial	heterorophic species	African clawed frog	present	yes	
**Reptiles --> Crocodylia**								
	Alligator mississisiensis	2	terrestrial	heterorophic species	Crocodilian	present	yes	
	Alligator sinensis	2	terrestrial	heterorophic species	Crocodilian	**absent**	yes	1
**Reptiles --> Squamata**								
	Gekko japonicus	2	terrestrial	heterorophic species	Geckos	**absent**	yes	6957
	Protobothrops mucrosquamatus	2	terrestrial	heterorophic species	Snakes	absent	yes	0
**Reptiles --> Chelonii**								
	Chelonia mydas	2	terrestrial	heterorophic species	Green sea turtle	present	yes	
	Chrysemys picta	2	terrestrial	heterorophic species	Painted turtle	present	yes	
**Birds**								
	Anas platyrhynchos	2	terrestrial	heterorophic species	Mallard (duck)	present	yes	
	Calidris pugnax	2	terrestrial	heterorophic species	Ruff	**absent**	yes	0
	Ficedula albicollis	2	terrestrial	heterorophic species	Collared flycatcher	present	yes	
	Gallus gallus	2	terrestrial	heterorophic species	Red junglefowl	present	yes	
	Lepidothrix coronata	2	terrestrial	heterorophic species	blue-crowned manakin	absent	yes	0
**Mammalian**								
	Callithrix jacchus	2	terrestrial	heterorophic species	Common marmoset	present	yes	
	Camelus dromedarius	2	terrestrial	heterorophic species	Dromedary	absent	yes	7
	Canis lupus	2	terrestrial	heterorophic species	Gray wolf	present	no	
	Ceratotherium simum	2	terrestrial	heterorophic species	White rhinoceros	present	yes	
	Chinchilla lanigera	2	terrestrial	heterorophic species	Long-tailed chinchilla	present	yes	
	Chrysochloris asiatica	2	terrestrial	heterorophic species	Cape golden mole	absent	yes	0
	Colobus angolensis	2	terrestrial	heterorophic species	Angola colobus	absent	yes	0
	Condylura cristata	2	terrestrial	heterorophic species	Star-nosed mole	absent	yes	0
	Dasypus novemcinctus	2	terrestrial	heterorophic species	Nine-banded armadillo	present	yes	
	Dipodomys ordii	2	terrestrial	heterorophic species	Kangaroo rats	present	yes	
	Elephantulus edwardii	2	terrestrial	heterorophic species	Cape elephant shrew	absent	yes	0
	Eptesicus fuscus	2	terrestrial	heterorophic species	Big brown bats	absent	yes	0
	Erinaceus europaeus	2	terrestrial	heterorophic species	European hedgehog	present	yes	
	Felis catus	2	terrestrial	heterorophic species	Cats	present	yes	
	Fukomys damarensis	2	terrestrial	heterorophic species	Damaraland mole-rat	present	yes	
	Galeopterus variegatus	2	terrestrial	heterorophic species	Sunda flying lemur	absent	yes	0
	Homo sapiens	2	terrestrial	heterorophic species	human	present	yes	
	Ictidomys tridecemlineatus	2	terrestrial	heterorophic species	Thirteen-lined ground squirrel	present	yes	
	Jaculus jaculus	2	terrestrial	heterorophic species	Jerboas	present	yes	
	Leptonychotes weddellii	2	marine	heterorophic species	Weddell seal	absent	yes	0
	Lipotes vexillifer	2	fresh-water	heterorophic species	Baiji	absent	yes	0
	Loxodonta africana	2	terrestrial	heterorophic species	African bush elephant	present	no	

	Melopsittacus undulatus	1	terrestrial	heterorophic species	Budgerigar	present	yes	
	Meriones unguiculatus	2	terrestrial	heterorophic species	Mongolian gerbil	absent	yes	1442
	Mesocricetus auratus	2	terrestrial	heterorophic species	Golden hamster	present	yes	
	Microcebus murinus	2	terrestrial	heterorophic species	Gray mouse lemur	present	yes	
	Microtus ochrogaster	2	terrestrial	heterorophic species	Prairie vole	present	yes	
	Monodelphis domestica	2	terrestrial	heterorophic species	Gray short-tailed opossum	present	yes	
	Mus musculus	2	terrestrial	heterorophic species	house mouse	present	yes	
	Mustela putorius furo	2	terrestrial	heterorophic species	Ferret	present	yes	
	Nomascus leucogenys	2	terrestrial	heterorophic species	Northern white-cheeked gibbon	present	yes	
	Octodon degus	2	terrestrial	heterorophic species	octodontid rodents	present	yes	
	Odobenus rosmarus	2	marine	heterorophic species	Walrus	present	yes	
	Orcinus orca	2	marine	heterorophic species	Killer whale	present	yes	
	Orycteropus afer	2	terrestrial	heterorophic species	Aardvark	present	yes	
	Otolemur garnettii	2	terrestrial	heterorophic species	Northern greater galago	present	yes	
	Ovis aries	2	terrestrial	heterorophic species	Sheep	present	yes	
	Pan paniscus	2	terrestrial	heterorophic species	Bonobo	present	yes	
	Pongo abelii	2	terrestrial	heterorophic species	Sumatran orangutan	present	yes	
	Propithecus coquereli	2	terrestrial	heterorophic species	Coquerel sifaka (lemur)	present	yes	
	Pteropus vampyrus	2	terrestrial	heterorophic species	Megabat	present	yes	
	Rattus norvegicus	2	terrestrial	heterorophic species	Brown rat	present	yes	
	Saimiri boliviensis	2	terrestrial	heterorophic species	Squirrel monkey	present	yes	
	Sus scrofa	2	terrestrial	heterorophic species	Wild boar	present	yes	
	Trichechus manatus	2	marine	heterorophic species	West Indian manatee	present	yes	
	Tupaia chinensis	2	terrestrial	heterorophic species	Tree shrew	present	yes	
**Bilateria --> Protostomia --> Molluscs**								
	Aplysia californica	2	marine	heterorophic species	California sea slug	present	yes	
	Biomphalaria glabrata	2	fresh water	heterorophic species	Snail	present	yes	
	Crassostrea gigas	2	marine	heterorophic species	Pacific oyster	present	yes	
	Haliotis discus	1	marine	heterorophic species	Sea snail	absent	yes	8154
	Haliotis diversicolor	1	marine	heterorophic species	Sea snail	absent	no	7435
	Meretrix meretrix	1	marine	heterorophic species	Saltwater clams	absent	yes	3131
	Mytilus galloprovincialis	1	marine	heterorophic species	Mediterranean mussel	absent	yes	19756
	Octopus bimaculoides	1	marine	heterorophic species	Octopus	present	yes	
	Pinctada fucata	1	marine	heterorophic species	Akoya pearl oyster	absent	yes	1380
**Bilateria --> Protostomia --> Platyhelminthes**								
	Clonorchis sinensis	0	terrestrial	animal parasites	human liver fluke	present	absent	
	Echinococcus granulosus	0	terrestrial	animal parasites	cyclophyllid cestode	present	absent	
	Echinococcus multilocularis	0	terrestrial	animal parasites	cyclophyllid taperworm	present	absent	

	Hymenolepis microstoma	0	terrestrial	animal parasites	rodent tapeworm	present	absent	
	Schistosoma haematobium	0	terrestrial	animal parasites	trematodes	present	absent	
	Schistosoma japonicum	0	terrestrial	animal parasites	trematodes	present	absent	
	Schistosoma mansoni	0	terrestrial	animal parasites	trematodes	present	absent	
	Schmidtea mediterranea	0	fresh water	heterorophic species	triclad	present	absent	
	Taenia solium	0	terrestrial	animal parasites	pork tapeworm	present	absent	
**Bilateria --> Protostomia --> Annelids**								
	Capitella teleta	2	marine	heterorophic species	annelid worm	present	yes	
	Helobdella robusta	1	fresh water	heterorophic species	leech	present	yes	
**Bilateria --> Protostomia --> Brachiopoda**								
	Lingula anatina	2	marine	heterorophic species		present	no	
**Bilateria --> Protostomia --> Rotifera**								
	Adineta vaga	0	fresh water	heterorophic species	bdelloid rotifer	present	absent	
**Bilateria --> Protostomia --> Tardigrades**								
	Hypsibius dujardini	0	terrestrial	heterorophic species	tardigrade	present	absent	
**Bilateria --> Protostomia --> Nematodes**								
**Classe I**								
	Trichuris muris	2	terrestrial	animal parasites	parasite of mice	present	yes	
	Trichuris suis	2	terrestrial	animal parasites	whipworm	present	yes	
**Classe III**								
	Acanthocheilonema viteae	2	terrestrial	animal parasites	rodent filarial worm	present	yes	
	Anisakis simplex	2	terrestrial	animal parasites	infect fish and marine mammals	present	yes	
	Ascaris lumbricoides	2	terrestrial	animal parasites	causes ascariasis	present	yes	
	Ascaris suum	2	terrestrial	animal parasites	causes ascariasis	present	yes	
	Brugia malayi	2	terrestrial	animal parasites	causes elephantiasis	present	yes	
	Brugia pahangi	2	terrestrial	animal parasites	nematode that infects domestic cats	present	yes	
	Brugia timori	3	terrestrial	animal parasites	human filarial parasitic nematode	present	yes	
	Dracunculus medinensis	2	terrestrial	animal parasites	causess dracunculiasis	present	yes	
	Elaeophora elaphi	2	terrestrial	animal parasites	parasitic roundworm	present	yes	
	Gongylonema pulchrum	3	terrestrial	animal parasites	human parasitic roundworms	present	yes	
	Loa loa	2	terrestrial	animal parasites	parasitic roundworm	present	yes	
	Onchocerca flexuosa	2	terrestrial	animal parasites	parasitic roundworm	present	no	
	Onchocerca ochengi	2	terrestrial	animal parasites	paraasitic roundworm	present	yes	
	Onchocerca volvulus	2	terrestrial	animal parasites	cause of river blindness in humans	present	yes	
	Ostertagia ostertagi	1	terrestrial	animal parasites	brown stomach worm	present	yes	
	Parascaris equorum	0	terrestrial	animal parasites	equine roundworm	absent	absent	17
	Thelazia callipaeda	2	terrestrial	animal parasites	cause thelaziasis	present	yes	
	Toxocara canis	2	terrestrial	animal parasites	dog roundworms	present	yes	
	Wuchereria bancrofti	2	terrestrial	animal parasites	human parasitic roundworms	present	yes	
**Classe IV**								
	Bursaphelenchus xylophilus	5	terrestrial	plant parasites	pine wood nematode	present	yes	
	Globodera pallida	0	terrestrial	plant parasites	plant pathogen (especially potatoes)	present	absent	
	Meloidogyne arenaria	3	terrestrial	plant parasites	peanut root knot nematode	present	yes	
	Meloidogyne floridensis	2	terrestrial	plant parasites	root-knot nematodes	present	yes	
	Meloidogyne hapla	3	terrestrial	plant parasites	root-knot nematodes	present	yes	

	Meloidogyne incognita	3	terrestrial	plant parasites	southern root-knot nematodes	present	yes	
	Meloidogyne javanica	4	terrestrial	plant parasites	tropical root-knot nematodes	present	yes	
	Panagrellus redivivus	3	terrestrial	heterorophic species	beer mat nematode	present	yes	
	Strongyloides papillosus	1	terrestrial	animal parasites	human pathogenic parasitic roundworm	present	yes	
	Strongyloides ratti	1	terrestrial	animal parasites	causess strongyloidiasis	present	yes	
	Strongyloides venezuelensis	1	terrestrial	animal parasites	threadworm, gastro-intestinal parasite in mouse	present	yes	
**Classe V**								
	Ancylostoma caninum	2	terrestrial	animal parasites	infects the small intestine of dogs	present	yes	
	Ancylostoma ceylanicum	3	terrestrial	animal parasites	zoonotic hookworm	present	yes	
	Ancylostoma duodenale	3	terrestrial	animal parasites	Old World hookworm	present	yes	
	Angiostrongylus cantonensis	2	terrestrial	animal parasites	roundworms that causes angiostrongyliasis	present	yes	
	Caenorhabditis angaria	3	terrestrial	heterorophic species	roundworms	present	yes	
	Caenorhabditis brenneri	3	terrestrial	heterorophic species	roundworms	present	yes	
	Caenorhabditis briggsae	4	terrestrial	heterorophic species	roundworms	present	yes	
	Caenorhabditis elegans	4	terrestrial	heterorophic species	roundworms	present	yes	
	Caenorhabditis japonica	3	terrestrial	heterorophic species	roundworms	present	yes	
	Caenorhabditis remanei	3	terrestrial	heterorophic species	roundworms	present	yes	
	Caenorhabditis sinica	3	terrestrial	heterorophic species	roundworms	present	yes	
	Caenorhabditis tropicalis	2	terrestrial	heterorophic species	roundworms	present	yes	
	Cylicostephanus goldi	2	terrestrial	animal parasites	animal parasites nematodes	present	no	
	Dictyocaulus viviparus	2	terrestrial	animal parasites	parasites of horses	present	yes	
	Dirofilaria immitis	2	terrestrial	animal parasites	heartworm or dog heartworm	present	yes	
	Haemonchus contortus	1	terrestrial	animal parasites	pathogenic nematodes of ruminants	present	yes	
	Haemonchus placei	2	terrestrial	animal parasites	parasitic roundworms	present	yes	
	Heligmosomoides polygyrus	3	terrestrial	animal parasites	intestinal roundworm of rodents	present	yes	
	Necator americanus	2	terrestrial	animal parasites	New World hookworm	present	yes	
	Nippostrongylus brasiliensis	1	terrestrial	animal parasites	gastrointestinal roundworm	present	yes	
	Oesophagostomum dentatum	2	terrestrial	animal parasites	human parasitc nematodes	present	no	
	Parastrongyloides trichosuri	0	terrestrial	animal parasites	an intestinal parasite of Australian Brush-tailed possums	present	absent	
	Pristionchus exspectatus	2	terrestrial	animal parasites	roundworms	present	yes	
	Pristionchus pacificus	2	terrestrial	animal parasites	roundworms	present	yes	
	Steinernema carpocapsae	4	terrestrial	animal parasites	entomopathogenic nematodes	present	yes	
	Steinernema feltiae	2	terrestrial	animal parasites	entomopathogenic nematodes	present	yes	
	Steinernema glaseri	1	terrestrial	animal parasites	entomopathogenic nematodes	present	yes	
	Steinernema monticolum	2	terrestrial	animal parasites	insect-parasitic nematode	present	yes	
	Steinernema scapterisci	2	terrestrial	animal parasites	mole cricket nematode	present	yes	

	Strongylus vulgaris	1	terrestrial	animal parasites	blood worm, common horse parasite	present	no	
	Teladorsagia circumcincta	2	terrestrial	animal parasites	nematodes that infect sheep and goats	present	yes	
**Classe Other chromadoera**								
	Enterobius vermicularis	1	terrestrial	animal parasites	Pinworm (enterobiasis)	present	yes	
	Romanomermis culicivorax	2	terrestrial	animal parasites	endoparasites in arthropods	present	yes	
	Syphacia muris	1	terrestrial	animal parasites	rat pinworm	present	yes	
	Trichinella nativa	1	terrestrial	animal parasites	nematode	absent	yes	16
	Trichinella spiralis	1	terrestrial	animal parasites	nematode	present	yes	
**Opisthokonts --> Metazoa --> Protostomia --> Arthropoda**								
**Crustacea**								
	Daphnia magna	2	fresh water	heterorophic species	small planctonic crustacean	absent	yes	15380
	Daphnia pulex	2	fresh water	heterorophic species	small planctonic crustacean	present	yes	
	Eriocheir sinensis	1	marine	heterorophic species	Chinese mitten crab	present	yes	
	Litopenaeus vannamei	1	marine	heterorophic species	Whiteleg shrimp	present	yes	
	Penaeus monodon	1	marine	heterorophic species	Asian tiger shrimp	present	yes	
	Pseudodiaptomus poplesia	1	marine	heterorophic species	a calanoid copepod	absent	yes	0
	Scylla paramamosain	2	marine	heterorophic species	mud and mangrove crab	present	yes	
**Chelicerata**								
	Amblyomma americanum	2	terrestrial	animal parasites	Lone Star tick	present	yes	
	Dermacentor variabilis	1	terrestrial	animal parasites	American dog ticks	present	yes	
	Haemaphysalis longicornis	1	terrestrial	animal parasites	ticks	present	yes	
	Ixodes scapularis	1	terrestrial	animal parasites	deer tick or black-legged tick	present	yes	
	Limulus polyphemus	1	marine	heterorophic species	Atlantic horseshoe crab	present	yes	
	Onychiurus arcticus	1	terrestrial	heterorophic species	Arctic springtail	present	yes	
	Rhipicephalus microplus	1	terrestrial	animal parasites	Asian blue tick	present	no	
	Sarcoptes scabiei	2	terrestrial	animal parasites	itch mites	present	yes	
	Stegodyphus mimosarum	1	terrestrial	heterorophic species	spiders	present	yes	
	Tetranychus urticae	1	terrestrial	plant parasites	red spider mite	present	yes	
**Myriapoda**								
	Strigamia maritima	2	marine	heterorophic species	European centipede	present	yes	
**Insecta --> Hymenoptera**								
	Acromyrmex echinatior	0	terrestrial	heterorophic species	New World ants	present	absent	
	Apis florea	0	terrestrial	heterorophic species	dwarf honey bee	present	absent	
	Apis mellifera	0	terrestrial	heterorophic species	western honey bee	present	absent	
	Atta cephalotes	0	terrestrial	heterorophic species	leafcutter ant	present	absent	
	Bombus impatiens	0	terrestrial	heterorophic species	common eastern bumble bee	present	absent	
	Bombus terrestris	0	terrestrial	heterorophic species	buff-tailed bumblebee	present	absent	
	Camponotus floridanus	0	terrestrial	heterorophic species	Florida capenter ant	present	absent	
	Harpegnathos saltator	0	terrestrial	heterorophic species	Indian jumping ant	present	absent	
	Linepithema humile	0	terrestrial	heterorophic species	Argentine ant	present	absent	
	Megachile rotundata	0	terrestrial	heterorophic species	alfalfa leafcutter bee	present	absent	
	Nasonia giraulti	0	terrestrial	heterorophic species	parasitoid	present	absent	

	Nasonia longicornis	0	terrestrial	heterorophic species	parasitoid	present	absent	
	Nasonia vitripennis	0	terrestrial	heterorophic species	parasitoid	present	absent	
	Pogonomyrmex barbatus	0	terrestrial	heterorophic species	red harvester ant	present	absent	
	Solenopsis invicta	0	terrestrial	heterorophic species	red imported fire ant	present	absent	
	Wasmannia auropunctata	0	terrestrial	heterorophic species	little fire ant	present	absent	
**Diptera**								
	Aedes aegypti	0	terrestrial	heterorophic species	yellow fever mosquito	present	absent	
	Aedes albopictus	0	terrestrial	heterorophic species	Asian tiger mosquito	present	absent	
	Anopheles albimanus	0	terrestrial	heterorophic species	mosquito	present	absent	
	Anopheles aquasalis	0	terrestrial	heterorophic species	mosquito	present	absent	
	Anopheles arabiensis	0	terrestrial	heterorophic species	mosquito	present	absent	
	Anopheles atroparvus	0	terrestrial	heterorophic species	mosquito	present	absent	
	Anopheles christyi	0	terrestrial	heterorophic species	mosquito	present	absent	
	Anopheles coluzzii	0	terrestrial	heterorophic species	mosquito	present	absent	
	Anopheles culicifacies	0	terrestrial	heterorophic species	mosquito	present	absent	
	Anopheles darlingi	0	terrestrial	heterorophic species	mosquito	present	absent	
	Anopheles dirus	0	terrestrial	heterorophic species	mosquito	present	absent	
	Anopheles epiroticus	0	terrestrial	heterorophic species	mosquito	present	absent	
	Anopheles farauti	0	terrestrial	heterorophic species	mosquito	present	absent	
	Anopheles funestus	0	terrestrial	heterorophic species	mosquito	present	absent	
	Anopheles gambiae	0	terrestrial	heterorophic species	mosquito	present	absent	
	Anopheles maculatus	0	terrestrial	heterorophic species	mosquito	present	absent	
	Anopheles melas	0	terrestrial	heterorophic species	mosquito	present	absent	
	Anopheles merus	0	terrestrial	heterorophic species	mosquito	present	absent	
	Anopheles minimus	0	terrestrial	heterorophic species	mosquito	present	absent	
	Anopheles quadriannulatus	0	terrestrial	heterorophic species	mosquito	present	absent	
	Anopheles sinensis	0	terrestrial	heterorophic species	mosquito	present	absent	
	Anopheles stephensii	0	terrestrial	heterorophic species	mosquito	present	absent	
	Clogmia albupunctata	0	terrestrial	heterorophic species	drain flies	present	absent	
	Culex quinquefasciatus	0	terrestrial	heterorophic species	southern house mosquito	present	absent	
	Drosophila albomicans	0	terrestrial	heterorophic species	fly	present	absent	
	Drosophila ananassae	0	terrestrial	heterorophic species	fly	present	absent	
	Drosophila biarmipes	0	terrestrial	heterorophic species	fly	present	absent	
	Drosophila bipectinata	0	terrestrial	heterorophic species	fly	present	absent	
	Drosophila elegans	0	terrestrial	heterorophic species	fly	present	absent	
	Drosophila erecta	0	terrestrial	heterorophic species	fly	present	absent	
	Drosophila eugracilis	0	terrestrial	heterorophic species	fly	present	absent	

	Drosophila ficusphila	0	terrestrial	heterorophic species	fly	present	absent	
	Drosophila grimshawi	0	terrestrial	heterorophic species	fly	present	absent	
	Drosophila kikkawai	0	terrestrial	heterorophic species	fly	present	absent	
	Drosophila melanogaster	0	terrestrial	heterorophic species	fly	present	absent	
	Drosophila miranda	0	terrestrial	heterorophic species	fly	present	absent	
	Drosophila mojavensis	0	terrestrial	heterorophic species	fly	present	absent	
	Drosophila neotestacea	0	terrestrial	heterorophic species	fly	present	absent	
	Drosophila persimilis	0	terrestrial	heterorophic species	fly	present	absent	
	Drosophila pseudoobscura	0	terrestrial	heterorophic species	fly	present	absent	
	Drosophila rhopaloa	0	terrestrial	heterorophic species	fly	present	absent	
	Drosophila santomea	0	terrestrial	heterorophic species	fly	present	absent	
	Drosophila sechellia	0	terrestrial	heterorophic species	fly	present	absent	
	Drosophila simulans	0	terrestrial	heterorophic species	fly	present	absent	
	Drosophila takahashii	0	terrestrial	heterorophic species	fly	present	absent	
	Drosophila virilis	0	terrestrial	heterorophic species	fly	present	absent	
	Drosophila willistoni	0	terrestrial	heterorophic species	fly	present	absent	
	Drosophila yakuba	0	terrestrial	heterorophic species	fly	present	absent	
	Episyrphus balteatus	0	terrestrial	heterorophic species	marmalade hoverfly	present	absent	
	Glossina austeni	0	terrestrial	heterorophic species	fly	present	absent	
	Glossina brevipalpis	0	terrestrial	heterorophic species	fly	present	absent	
	Glossina fuscipes	0	terrestrial	heterorophic species	fly	present	absent	
	Glossina morsitans morsitans	0	terrestrial	heterorophic species	fly	present	absent	
	Glossina pallidipes	0	terrestrial	heterorophic species	Tsetse fly	present	absent	
	Lutzomyia longipalpis	0	terrestrial	heterorophic species	blood sucker	present	absent	
	Mayetiola destructor	0	terrestrial	heterorophic species	Hessian fly or barley midge	present	absent	
	Megaselia scalaris	0	terrestrial	heterorophic species	fly	present	absent	
	Musca domestica	0	terrestrial	heterorophic species	housefly	present	absent	
	Phlebotomus papatasi	0	terrestrial	heterorophic species	sand flies	present	absent	
	Stomoxys calcitrans	0	terrestrial	heterorophic species	stable fly, dog fly	present	absent	
	Triatoma infestans	0	terrestrial	heterorophic species	winchuka, "kissing bug", blood sucker	absent	absent	3488
**Hemiptera**								
	Acyrthosiphon kondoi	2	terrestrial	plant parasites	aphid	absent	yes	23.041
	Acyrthosiphon pisum	4	terrestrial	plant parasites	pea aphid	present	yes	
	Aphis gossypii	2	terrestrial	plant parasites	melon and cotton aphid	present	yes	
	Cimex lectularius	0	terrestrial	free-livng species	Bed bugs - human primary hosts	present	absent	
	Diaphorina citri	1	terrestrial	plant parasites	Asian citrus psyllid	present	yes	
	Maconellicoccus hirsutus	1	terrestrial	plant parasites	pink, grape or hibiscus mealybug	absent	yes	7.696

	Myzus ascalonicus	3	terrestrial	plant parasites	Shallot aphid	absent	yes	22.942
	Myzus persicae	3	terrestrial	plant parasites	Green peach aphid	present	yes	
	Nilaparvata lugens	1	terrestrial	plant parasites	brown planthopper	absent	yes	118.141
	Peregrinus maidis	1	terrestrial	plant parasites	Corn planthopper	absent	yes	20.678
	Rhodnius prolixus	0	terrestrial	free-livng species	principal triatomine vector of the Chagas (trypanosoma cruzi) parasite	present	absent	
	Rhopalosiphum maidis	3	terrestrial	plant parasites	Corn leaf aphid	present	yes	
	Rhopalosiphum padi	3	terrestrial	plant parasites	Bird cherry-oat aphid	present	yes	
	Sitobion avenae	1	terrestrial	plant parasites	English grain aphid	absent	yes	3.614
**Lepidoptera**								
	Agrotis segetum	1	terrestrial	heterorophic species	turnip moth	present	yes	
	Amyelois transitella	3	terrestrial	heterorophic species	navel orangeworm	present	yes	
	Antheraea assama	1	terrestrial	heterorophic species	muga silkworm	absent	yes	35,191
	Antheraea mylitta	1	terrestrial	heterorophic species	tasar silkworm	absent	yes	3.913
	Antheraea yamamai	1	terrestrial	heterorophic species	Japanese silk moth	absent	yes	662
	Bicyclus anynana	1	terrestrial	heterorophic species	squinting bush brown	absent	yes	101.595
	Bombyx mori	2	terrestrial	heterorophic species	domestic silkmoth	present	yes	
	Danaus plexippus	2	terrestrial	heterorophic species	monarch butterfly	present	yes	
	Heliconius melpomene	3	terrestrial	heterorophic species	postman butterfly	present	yes	
	Helicoverpa armigera	1	terrestrial	heterorophic species	cotton bullworm, corn earworm	present	yes	
	Heliothis virescens	1	terrestrial	heterorophic species	tobacco budworm	present	yes	
	Manduca sexta	1	terrestrial	heterorophic species	Carolina sphinx moth	present	yes	
	Melitaea cinxia	2	terrestrial	heterorophic species	Glanville fritillary butterfly	present	yes	
	Ostrinia nubilasis	1	terrestrial	heterorophic species	European corn borer	absent	yes	15.040
	Papilio machaon	2	terrestrial	heterorophic species	Old World swallowtail butterfly	present	yes	
	Papilio polytes	2	terrestrial	heterorophic species	common mormon butterfly	present	yes	
	Papilio xuthus	2	terrestrial	heterorophic species	Asian swallowtail butterfly	present	yes	
	Plutella xylostella	1	terrestrial	heterorophic species	diamondback moth, cabbage moth	present	yes	
	Spodoptera exigua	1	terrestrial	heterorophic species	armyworms pests	absent	yes	16359
	Spodoptera frugiperda	2	terrestrial	heterorophic species	armyworms pests	present	yes	
	Spodoptera littoralis	1	terrestrial	heterorophic species	armyworms pests	absent	yes	72.261
	Trichoplusia ni	1	terrestrial	heterorophic species	cabbage looper, pests	present	yes	
**Phthiraptera**								
	Pediculus humanus	0	terrestrial	heterorophic species	head louse, ectoparasite of humans	present	yes	
**Coleoptera**								
	Dendroctonus ponderosae	2	terrestrial	heterorophic species	mountain pine beetle	present	yes	
	Pissodes strobi	1	terrestrial	plant parasites	white pine weevil	absent	yes	69.475
	Sitophilus oryzae	1	terrestrial	plant parasites	rice weevil	absent	yes	25.748
	Sphenophorus levis	1	terrestrial	plant parasites	weevil	absent	yes	3.447
	Tribolium castaneum	1	terrestrial	heterorophic species	red flour beetle	present	yes	
**Orthoptera**								

	Gryllus bimaculatus	1	terrestrial	heterorophic species	cricket	present	yes	
	Schistocerca gregaria	1	terrestrial	heterorophic species	locust	absent	yes	34.709
**Blattoptera**								
	Coptotermes formosanus	1	terrestrial	heterorophic species	formosan subterranean termite	absent	yes	142.814
	Periplaneta americana	1	terrestrial	heterorophic species	america cockroach	absent	yes	5.394
	Reticulitermes flavipes	1	terrestrial	heterorophic species	eastern subterranean termite	absent	yes	23.767
	Zootermopsis nevadensis	1	terrestrial	heterorophic species	eusocial dampwood termite	present	yes	
**Archaeplastida --> Glaucophytes**								
	Cyanophora paradoxa	3	fresh water	autotrophic species	algaes, model organism	present	no	
**Rhodophytes**								
	Chondrus crispus	0	marine	autotrophic species	red algaes called Irish moss or carageen moss	present	absent	
	Cyanidioschyzon merolae	1	marine	autotrophic species	red algaes	present	yes	
	Galdieria sulphuraria	1	marine	autotrophic species	extermophilic red algaes	present	yes	
	Grateloupia livida	1	marine	autotrophic species	red algaes	present	yes	
	Porphyridium purpureum	1	marine	autotrophic species	red algaes	present	yes	
**Green algae - Chlorophyta**								
	Alnus glutinosa	1	terrestrial	autotrophic species	black alder	absent	yes	32620
	Chlamydomonas reinhardtii	1	marine	autotrophic species	green algaes	present	yes	
	Chlorella variabilis	1	marine	autotrophic species	green algaes	present	yes	
	Gonium pectorale	1	marine	autotrophic species	colonial algaes	absent	yes	0
	Klebsormidium flaccidum	1	marine	autotrophic species	filamentous charophyte green algaes	present	yes	
	Monoraphidium neglectum	1	marine	autotrophic species	green algaes	present	yes	
	Volvox carteri	1	marine	autotrophic species	colonial green algaes	present	yes	
**Moss - Bryophyta**								
	Physcomitrella patens	1	terrestrial	autotrophic species	moss	present	yes	
**Ferns**								
	Acrostichum aureum	1	terrestrial	autotrophic species	golden leather fern	absent	yes	0
	Acrostichum speciosum	1	terrestrial	autotrophic species	Mangrove fern	absent	yes	0
	Adiantum capillus	1	terrestrial	autotrophic species	black maidenhair fern	absent	yes	30544
	Azolla filiculoides	1	fresh water	autotrophic species	floating aquatic fern	present	yes	
	Ceratopteris richardii	1	terrestrial	autotrophic species	fern	absent	yes	5133
	Ceratopteris thalictroides	1	terrestrial	autotrophic species	water sprite, Indian fern	absent	yes	0
	Lygodium japonicum	1	terrestrial	autotrophic species	vine-like fern or Japanese climbing fern	absent	yes	0
	Pteridium aquilinum	1	terrestrial	autotrophic species	eagel fern	absent	yes	424
**Ferns - relative**								
	Selaginella moellendorffii	4	terrestrial	autotrophic species	model organism	present	yes	
**Gymnospermes**								
	Picea sitchensis	2	terrestrial	autotrophic species	sitka spruce	present	yes	
**Marchantiales**								
	Marchantia polymorpha	1	terrestrial	autotrophic species	umbrella liverwort	present	yes	

Angiospermes - Dicotylédons								
	Alnus glutinosa	1	terrestrial	autotrophic species	black alder		yes	
	Amborella trichopoda	2	terrestrial	autotrophic species	understory shrubs		yes	
	Arabidopsis lyrata	3	terrestrial	autotrophic species	thale cress, mouse-ear cress	present	yes	
	Arabidopsis thaliana	3	terrestrial	autotrophic species	thale cress, mouse-ear cress	present	yes	
	Artemisia annua	1	terrestrial	autotrophic species	sweet wormwood		yes	
	Beta vulgaris	4	terrestrial	autotrophic species	sugar beet, spinach beet	present	yes	
	Botryococcus braunii	1	marine	autotrophic species	green, pyramid-shaped planktonic microalgua	present	no	
	Brachypodium distachyon	3	terrestrial	autotrophic species	purple false brome	present	yes	
	Brassica napus	3	terrestrial	autotrophic species	oilseed rape	present	yes	
	Brassica oleracea	4	terrestrial	autotrophic species	wild cabbage plants	present	yes	
	Camelina sativa	3	terrestrial	autotrophic species	gold-of-pleasure or false flax	present	yes	
	Camellia sinensis	1	terrestrial	autotrophic species	tea plant	present	no	
	Catharanthus roseus	1	terrestrial	autotrophic species	Madagascar periwinkle	present	no	
	Cicer arietinum	3	terrestrial	autotrophic species	chickpea	present	yes	
	Citrus clementina	3	terrestrial	autotrophic species	clementine	present	yes	
	Citrus reticulata	1	terrestrial	autotrophic species	mandarin orange	present	no	
	Coffea canephora	3	terrestrial	autotrophic species	robusta coffee	present	yes	
	Cucumis melo	3	terrestrial	autotrophic species	muskmelon	present	yes	
	Dendrobium nobile	1	terrestrial	autotrophic species	member of Orchidaceae	present	no	
	Dioscorea alata	1	terrestrial	autotrophic species	purple yam	present	no	
	Erythranthe guttata	3	terrestrial	autotrophic species	seep monkey flower	present	yes	
	Eucalyptus grandis	3	terrestrial	autotrophic species	flooded gum or rose gum	present	yes	
	Euonymus alatus	1	terrestrial	autotrophic species	winged spindle	present	no	
	Eutrema salsugineum	3	terrestrial	autotrophic species	rockcress	present	yes	
	Fragaria vesca	3	terrestrial	autotrophic species	strawberry	present	yes	
	Glycine max	3	terrestrial	autotrophic species	soybean	present	yes	
	Gossypium raimondii	3	terrestrial	autotrophic species	cotton plant	present	yes	
	Jatropha curcas	3	terrestrial	autotrophic species	physic nut, Barbados nut, poison nut, bubble bush or purging nut	present	yes	
	Lotus japonicus	3	terrestrial	autotrophic species	wild legume	present	yes	
	Malus domestica	3	terrestrial	autotrophic species	apple	present	yes	
	Medicago truncatula	4	terrestrial	autotrophic species	barrelclover	present	yes	
	Nelumbo nucifera	3	terrestrial	autotrophic species	Indian lotus	present	yes	
	Nicotiana sylvestris	4	terrestrial	autotrophic species	common domesticated tobacco	present	yes	
	Nicotiana tomentosiformis	2	terrestrial	autotrophic species	tobacco	present	no	
	Panax ginseng	1	terrestrial	autotrophic species	ginseng	present	no	

	Panicum virgatum	1	terrestrial	autotrophic species	swichgrass	present	no	
	Phaseolus vulgaris	3	terrestrial	autotrophic species	common bean	present	yes	
	Populus euphratica	4	terrestrial	autotrophic species	Euphrates poplar or desert poplar	present	yes	
	Populus trichocarpa	2	terrestrial	autotrophic species	black cottonwood	present	yes	
	Prunus mume	3	terrestrial	autotrophic species	Chinese plum or Japanese apricot	present	yes	
	Pyrus bretschneideri	3	terrestrial	autotrophic species	Chinese white pear	present	yes	
	Rehmannia glutinosa	1	terrestrial	autotrophic species	use in Chinese traditional medicine	absent	no	1301
	Ricinus communis	3	terrestrial	autotrophic species	castorbean or castor-oil-plant	present	yes	
	Sesamum indicum	3	terrestrial	autotrophic species	sesame	present	yes	
	Solanum lycopersicum	4	terrestrial	autotrophic species	tomato	present	yes	
	Solanum tuberosum	3	terrestrial	autotrophic species	potato	present	yes	
	Tarenaya hassleriana	4	terrestrial	autotrophic species	cleome hassleriana, spider flower or pink queen	present	yes	
	Theobroma cacao	3	terrestrial	autotrophic species	cacao or cocoa tree	present	yes	
	Triticum aestivum	3	terrestrial	autotrophic species	bread wheat	present	yes	
	Triticum urartu	1	terrestrial	autotrophic species	red wild einkorn	present	yes	
	Vigna radiata	4	terrestrial	autotrophic species	mung bean	present	yes	
	Vitis vinifera	4	terrestrial	autotrophic species	grape vine	present	yes	
	Wolffia arrhiza	1	terrestrial	autotrophic species	spotless watermeal and rootless duckweed	present	no	
**Angiospermes - Monocotylédons Liliopsida**								
	Brachypodium distachyon	3	terrestrial	autotrophic species	purple false brome or stiff brome	present	yes	
	Elaeis guineensis	2	terrestrial	autotrophic species	oil palm or macaw-fat	present	yes	
	Hordeum vulgare	2	terrestrial	autotrophic species	barley	present	yes	
	Musa acuminata	3	terrestrial	autotrophic species	banana species	present	yes	
	Oryza brachyantha	3	terrestrial	autotrophic species	grass in the rice genus	present	yes	
	Phoenix dactylifera	3	terrestrial	autotrophic species	date palm	present	yes	
	Setaria italica	3	terrestrial	autotrophic species	poales, foxtail millet	present	yes	
	Zea mays	3	terrestrial	autotrophic species	maize	present	yes	
	Zostera marina	2	marine	autotrophic species	common eelgrass, seawrack	present	yes	
