# Supplementary material for: Cross-Kingdom Analysis of Diversity, Evolutionary History, and Site Selection within the Eukaryotic Macrophage Migration Inhibitory Factor Superfamily
Source: Genes (Basel). 2019 Sep 24;10(10):740. doi: 10.3390/genes10100740 (PMC6826473; doi:10.3390/genes10100740)
Supplement: Supplementary file 1 [file genes-10-00740-s001.zip › Michelet suppl/table S2 Genes┬á.docx]

Supplementary table S2: Species and accession numbers of MIF sequences used as queries for the BLAST searches

| Species name | Accession numbers |
| --- | --- |
| *Homo sapiens* | Genbank accession numbers NP_002406.1 and NP_00107786 |
| *Biomphalaria glabrata* | Genbank accession numbers: XP_013094629.1 and ACR81565.1 |
| *Ancylostoma caninum* | Wormbase parasite accession numbers: ANCCAN_11528 and ANCCAN_25101 |
| *Plasmodium berghei* | Ensembl accession number : CDS52161 |
| *Leishmania major* | Ensembl accession numbers: LmjF.33.1740 and LmjF.33.1750 |
| *Arabidopsis thaliana* | Genbank accession numbers: NP_200527.1, NP_195785.1 and NP_566955.1 |
